
JOURNAL INFORMATION
==============================
NLM Title Abbreviation: Access Microbiol
Journal ID: Access Microbiol
NLM Journal ID: 101746927
Journal ID: acmi

Access Microbiology

EISSN: 2516-8290
Publisher: Microbiology Society

ARTICLE INFORMATION
==============================
PMCID: PMC7702993
PMID: 33324866
Article version: 1
Subjects: Oral Abstract

ACMI conference posters 2019

Publication date: 2019
Electronic publication date: 2019 Apr 8
Volume: 1
Issue: 1A
Electronic Location ID: 15
Copyright: © 2019 The Authors
Copyright year: 2019
License: This is an open-access article distributed under the terms of the Creative Commons Attribution License.
License URL: https://creativecommons.org/licenses/by/4.0/

==============================


Viral hijacking of the nucleolar DNA-damage response machinery: a novel mechanism to regulate host cell biology

http://jmmcr.microbiologyresearch.org

Rawlinson Stephen M 1
Zhao Tianyue 1
Rozario Ashley M. 1
Rootes Christina L. 2
McMillan Paul J. 3
Purcell Anthony W. 1
Woon Amanda 1
Marsh Glenn A. 1
Lieu Kim G. 1
Wang Lin-Fa 4
Netter Hans J. 5
Bell Toby D. M. 1
Stewart Cameron R. 2
Moseley Gregory W. 1 *
1 Monash University , Melbourne , Australia
2 CSIRO , Geelong , Australia
3 University of Melbourne , Melbourne , Australia
4 Duke-NUS , Singapore , Singapore
5 Victorian Infectious Diseases Reference Laboratory , Melbourne , Australia
*Correspondence: Greg Moseley, greg.moseley@monash.edu

Microbiological profile and risk factors for in-hospital mortality of infective endocarditis intertiary care hospitals of south Vietnam

http://jmmcr.microbiologyresearch.org

Tran Hoang *
Pham Ngoc Thach University of Medicine , Ho Chi Minh City , Vietnam
*Correspondence: Hoang Tran, bacsitranminhhoang@gmail.com

The effect of epinephrine and norepinephrine on the growth and pathogenicity of Campylobacter jejuni

http://jmmcr.microbiologyresearch.org

Truccollo Brendha 1 2 *
Whyte Paul 2
Bolton Declan J. 1
1 Teagasc Food Research Centre , Dublin , Ireland
2 University College Dublin , Dublin , Ireland
*Correspondence: Brendha Truccollo, brendha.truccollo@teagasc.ie

In-ovo antiviral assay of methanolic leaf extract of Cymbopogon citratus (Lemon grass) on Newcastle disease virus

http://jmmcr.microbiologyresearch.org

Abraham-Oyiguh Joseph 1 2 *
Zakka Abigail W. 2
Onwuatuegwu Joseph Taiwo C. 3
Sulaiman Lanre K. 4
Muhammad Salamatu Y. 2
1 Federal Polytechnic Idah , Kogi State , Nigeria
2 Kaduna State University , Kaduna , Nigeria
3 Tansian University , Umunya , Nigeria
4 National Verinary Research Institute , Vom, Jos , Nigeria
*Correspondence: Joseph Abraham-Oyiguh, josephoyiguh@yahoo.com
Keywords: Cymbopogon citratus, Newcastle disease virus, Phytochemical, embryonated hens eggs, Spot haemagglutination, Methanol extract

Mathematical modelling to characterise the in vivo dynamics of Salmonella in the naïve and immunised host

http://jmmcr.microbiologyresearch.org

Vlazaki Myrto *
Restif Olivier
University of Cambridge , Cambridge , United Kingdom
*Correspondence: Myrto Vlazaki, mv382@cam.ac.uk

Structural elucidation of viral antagonism of innate immunity: the STAT1 interface

http://jmmcr.microbiologyresearch.org

Moseley Gregory W. 1 *
Hossain Alamgir 2
Larrous Florence 3
Zhan Jingyu 2
Sethi Ashish 2
Ibrahim Youssef 2
Aloi Maria 1
Rawlinson Stephen M. 1
Lieu Kim G. 2
Mok Yee-Foong 2
Griffin Michael D. W. 2
Ito Naoto 4
Ose Toyoyuki 5
Bourhy Hervé 3
Gooley Paul R. 2
1 Monash University , Melbourne , Australia
2 University of Melbourne , Melbourne , Australia
3 Pasteur Institute , Paris , France
4 Gifu University , Gifu , Japan
5 Hokkaido University , Sapporo , Japan
*Correspondence: Greg Moseley, greg.moseley@monash.edu

Shining new lights on chytrid cell biology: quantitative live cell imaging of rhizoid development in an early-diverging fungus

http://jmmcr.microbiologyresearch.org

Laundon Davis 1 2 *
Wheeler Glen 1
Mock Thomas 2
Cunliffe Michael 1 3
1 The Marine Biological Association , Plymouth , United Kingdom
2 University of East Anglia , Norwich , United Kingdom
3 Plymouth University , Plymouth , United Kingdom
*Correspondence: Davis Laundon, davlau@mba.ac.uk

The HamE scaffold positively regulates MpkB phosphorylation to promote development and secondary metabolism in Aspergillus nidulans

http://jmmcr.microbiologyresearch.org

Frawley Dean *
Karahoda Betim
Bayram Ozlem Sarikaya
Bayram Ozgur
Maynooth University , Maynooth , Ireland
*Correspondence: Dean Frawley, dean_frawley@hotmail.com

Identification of Acinetobacter baumannii type VI secretion system effectors and characterisation of a novel effector/immunity pair

http://jmmcr.microbiologyresearch.org

Lewis Jessica *
Lucas Deanna Deveson
Harper Marina
Boyce John
Monash Biomedicine Discovery Institute, Monash University , Clayton , Australia
*Correspondence: Jessica Lewis, jessica.lewis@monash.edu

Inter-species transmission of avian influenza virus to dogs: 10 years experience

http://jmmcr.microbiologyresearch.org

Song Daesub *
Na Woonsung
Yeom Minjoo
Korea University , Sejong, Korea , Republic of
*Correspondence: Daesub Song, sds1@korea.ac.kr

Understanding the killing mechanism of action by virus-infected yeasts

http://jmmcr.microbiologyresearch.org

Dikicioglu Duygu *
University of Cambridge Department of Chemical Engineering and Biotechnology , Cambridge , United Kingdom
*Correspondence: Duygu Dikicioglu, dd345@cam.ac.uk

Regulation of exploratory growth and antibiotic production in Streptomyces venezuelae by the two-component system CutRS

http://jmmcr.microbiologyresearch.org

McLean Thomas C. 1 *
Wilkinson Barrie 2
Hutchings Matthew I. 1
1 University of East Anglia , Norwich , United Kingdom
2 John Innes Centre , Norwich , United Kingdom
*Correspondence: Thomas McLean, t.mclean@uea.ac.uk

Study on seroprevalence of IgG antibody of Varicella-Zoster virus in Pregnant mothers, Neonates&childrens of six month age

http://jmmcr.microbiologyresearch.org

Yasmin Selina *
Chowdhury Mosharof Hossain Prof. Dr. Md.
Sylhet Women’s Medical College , Sylhet , Bangladesh
*Correspondence: Selina Yasmin, drselinayasmin@gmail.com

Redefining a new genomic blueprint of the human gut microbiota

http://jmmcr.microbiologyresearch.org

Almeida Alexandre 1 2 *
Mitchell Alex 1
Boland Miguel 1
Forster Samuel 3 4
Gloor Gregory 5
Tarkowska Aleksandra 1
Thomson Nicholas 2
Lawley Trevor 2
Finn Robert 1
1 EMBL-EBI , Hinxton , United Kingdom
2 Wellcome Sanger Institute , Hinxton , United Kingdom
3 CiiiD , Clayton , Australia
4 Monash University , Clayton , Australia
5 University of Western Ontario , London , Canada
*Correspondence: Alexandre Almeida, aalmeida@ebi.ac.uk

When the metabolic model says NO: untangling the Gordian knot of TB’s intracellular metabolism

http://jmmcr.microbiologyresearch.org

Basu Piyali 1
Sandhu Noor 1
Bhatt Apoorva 2
Singh Albel 2
Balhana Ricardo 1
Gobe Irene 1
Crowhurst Nicola 1
Mendum Tom 1
Gao Liang 3
Ward Jane 3
Beale Mike 3
McFadden Johnjoe 1
Beste Dany 1 *
1 University of Surrey , Guildford , United Kingdom
2 University of Birmingham , Birmingham , United Kingdom
3 Rothamsted Institute , Rothamsted , United Kingdom
*Correspondence: Dany Beste, d.beste@surrey.ac.uk

The chemical ecology of protective microbiomes in plant roots and leafcutter ants

http://jmmcr.microbiologyresearch.org

Worsley Sarah *
Hutchings Matt
Murrell Colin
University of East Anglia , Norwich , United Kingdom
*Correspondence: Sarah Worsley, s.worsley@uea.ac.uk

Rabies virus: defining antigenic requirements for pan-lyssavirus neutralisation

http://jmmcr.microbiologyresearch.org

Shipley Rebecca 1 2 *
Selden David 2
Guanghui Wu 2
Wright Edward 1
Fooks Anthony R 2 3 4
Banyard Ashley C 2
1 University of Sussex , Falmer , United Kingdom
2 Animal and Plant Health Agency (APHA) , Weybridge , United Kingdom
3 St. George’s Hospital Medical School , London , United Kingdom
4 University of Liverpool , Liverpool , United Kingdom
*Correspondence: Rebecca Shipley, rebecca_shipley@live.com

iPSCs derived from endothelial progenitors model latency and reactivation during human cytomegalovirus infection

http://jmmcr.microbiologyresearch.org

Poole Emma 1 *
Huang Chris 1
Forbester Jessica 1
Shnayder Miri 2
Nachson Aharon 2
Kweider Baraa 1
Basaj Anna 1
Smith Daniel 1
Jackson Sarah 1
Kiskin Fedir 1
Roche Kate 3
Murphy Eain 3
Wills Mark 1
Dougan Gordon 1
Stern-Ginossar Noam 2
Rana Amer 1
Sinclair John 1
1 Cambridge University , Cambridge , United Kingdom
2 Weizmann , Rehovat , Israel
3 Cleveland , Ohio , USA
*Correspondence: Emma Poole, elp27@cam.ac.uk

Hydrogenotrophic methanogenesis dominates at high pH

http://jmmcr.microbiologyresearch.org

Wormald Richard *
Humphreys Paul
University of Huddersfield , Huddersfield , United Kingdom
*Correspondence: Richard Wormald, Richard.Wormald2@hud.ac.uk

Epidemiology of tick borne pathogens of dogs in Nigerian communities

http://jmmcr.microbiologyresearch.org

Apaa Ternenge Thaddaeus 1 2 *
Dunham Stephen 1
Tarlinton Rachael 1
1 University of Nottingham , Nottingham , United Kingdom
2 University of Agriculture Makurdi , Benue State, Makurdi , Nigeria
*Correspondence: Ternenge Thaddaeus Apaa, docapath@yahoo.com

The MtrAB-LpqB two component system links development with secondary metabolite production in Streptomyces species and can be manipulated to switch on silent secondary metabolite clusters

http://jmmcr.microbiologyresearch.org

Som Nicolle F. 1 *
Heine Daniel 2
Holmes Neil A. 1
Munnoch John T. 3
Knowles Felicity 4
Chandra Govind 5
Seipke Ryan F. 6
Hoskisson Paul A. 7
Wilkinson Barrie 5
Hutchings Matthew I. 1
1 University of East Anglia , Norwich , United Kingdom
2 BASF SE , Ludwigshafen , Germany
3 Strathclyde Institute of Pharmacy and Biomedical Sciences , Glasgow , United Kingdom
4 Earlham Institute , Norwich , United Kingdom
5 John Innes Centre , Norwich , United Kingdom
6 The Astbury Centre for Structural Molecular Biology , Leeds , United Kingdom
7 Strat Strathclyde Institute of Pharmacy and Biomedical Scienceshclyde Institute of Pharmacy and Biomedical Sciences , Glasgow , United Kingdom
*Correspondence: Nicolle Som, n.som@uea.ac.uk

Marine fungal dark matter in the global ocean

http://jmmcr.microbiologyresearch.org

Chrismas Nathan *
Cunliffe Michael
Marine Biological Association of the UK , Plymouth , United Kingdom
*Correspondence: Nathan Chrismas, natchr@mba.ac.uk

The Sodalis system and flux balance analysis as a tool for investigating insect-microbe interactions and the evolution of symbioses

http://jmmcr.microbiologyresearch.org

Hall Rebecca 1 *
Flanagan Lindsey 1
Bottery Michael 1
Springthorpe Vicki 1
Thorpe Stephen 1
Darby Alistair 2
Wood Jamie 1
Thomas Gavin 1
1 University of York , York , United Kingdom
2 University of Liverpool , Liverpool , United Kingdom
*Correspondence: Rebecca Hall, rjh526@york.ac.uk

Understanding aurodox: A Type III secretion system inhibitor from Streptomyces goldiniensis

http://jmmcr.microbiologyresearch.org

McHugh Rebecca Elizabeth 1 2 *
1 Strathclyde Institute of Pharmacy and Biomedical Sciences, University of Strathclyde , Glasgow , United Kingdom
2 Institute of Infection, Immunity and Inflammation, University of Glasgow , Glasgow , United Kingdom
*Correspondence: Rebecca McHugh, rebecca.mchugh@strath.ac.uk

Investigating the role of nitric oxide in plant root colonisation by Streptomyces spp.

http://jmmcr.microbiologyresearch.org

Newitt Jake 1 *
Munnoch John 2
Worsley Sarah 1
Hutchings Matt 1
1 University of East Anglia , Norwich , United Kingdom
2 University of Strathclyde , Glasgow , United Kingdom
*Correspondence: Jake Newitt, jake.newitt@outlook.com

Surfactants from the sea: rhamnolipid production by marine bacteria

http://jmmcr.microbiologyresearch.org

Twigg Matthew 1 *
Tripathi Lakshmi 1
Zompra Katerina 2
Salek Karina 3
Irorere Victor 1
Gutierrez Tony 3
Spyroulias Georgios 2
Marchant Roger 1
Banat Ibrahim 1
1 Ulster University , Coleraine , United Kingdom
2 University of Patras , Patras , Greece
3 Heriot-Watt University , Edinburgh , United Kingdom
*Correspondence: Matthew Twigg, m.twigg@ulster.ac.uk

Discovering novel antimicrobials from Streptomyces formicae, a symbiont of fungus farming plant ants, using CRISPR/Cas9 genome editing

http://jmmcr.microbiologyresearch.org

Devine Rebecca 1 *
Qin Zhiwei 2
Wilkinson Barrie 2
Hutchings Matt 1
1 University of East Anglia , Norwich , United Kingdom
2 John Innes Centre , Norwich , United Kingdom
*Correspondence: Rebecca Devine, R.Devine@uea.ac.uk

Identification of novel host factors influencing human cytomegalovirus replication using a two-step siRNA screen

http://jmmcr.microbiologyresearch.org

Lee Chen-Hsuin 1 *
Griffiths Samantha 2
Digard Paul 1
Pham Nhan 2
Auer Manfred 2
Haas Juergen 2
Grey Finn 1
1 Roslin Institute, University of Edinburgh , Edinburgh , United Kingdom
2 University of Edinburgh , Edinburgh , United Kingdom
*Correspondence: Chen-Hsuin Lee, abraham.lee@roslin.ed.ac.uk

In-depth profiling of calcite precipitation by environmental bacteria reveals fundamental mechanistic differences with relevance to application

http://jmmcr.microbiologyresearch.org

Reeksting Bianca *
Paine Kevin
Gebhard Susanne
University of Bath , Bath , United Kingdom
*Correspondence: Bianca Reeksting, bjreeksting@gmail.com

Illuminating the molecular choreography of multi-segmented RNA genomes

http://jmmcr.microbiologyresearch.org

Borodavka Alex 1 2 3 *
Strauss Sebastian 3
Coria Aaztli 4
Dykeman Eric 5
Jungmann Ralf 3
Lamb Don 2
1 University of Leeds , Leeds , United Kingdom
2 Ludwig-Maximilians-Universität , Munich , Germany
3 Max Planck Institute of Biochemistry , Munich , Germany
4 University of North Carolina , Chapel Hill , USA
5 University of York , York , United Kingdom
*Correspondence: Alexander Borodavka, a.borodavka@leeds.ac.uk

The effect of antibiotic and nutrient limitation to antibiotic resistant bacteria in single-cell level

http://jmmcr.microbiologyresearch.org

Liu Li 1 *
Kreft Jan 2
Vigolo Daniele 2
1 Beihang University , Beijing , China
2 University of Birmingham , Birmingham , United Kingdom
*Correspondence: Li Liu, lililiu@buaa.edu.cn

Optimisation of an in vitro cell system using pseudoviruses to investigate HBV entry mechanisms

http://jmmcr.microbiologyresearch.org

Goddard Chun *
Irving William
Tarr Alexander
The University of Nottingham , Nottingham , United Kingdom
*Correspondence: Chun Goddard, chun.goddard@nottingham.ac.uk

HIV-1 Vpr accessory protein interacts with REAF and mitigates its associated anti-viral activity

http://jmmcr.microbiologyresearch.org

Gibbons Joseph 1
Marno Kelly 1
Pike Rebecca 1
Lee Jason 1
Jones Christopher 1
Ogunkolade William 1
Pardieu Claire 1
Warnes Gary 1
Rowley Paul 2
Sloan Richard 1
McKnight Aine 1 *
1 Queen Mary University of London, Bart’s and The London School of Medicine and Dentistry , London , United Kingdom
2 University of Idaho , Moscow , USA
*Correspondence: Aine McKnight, a.mcknight@qmul.ac.uk

Signal-integration through PhoPQ enables Salmonella to adapt to heterogeneous tissue microenvironments

http://jmmcr.microbiologyresearch.org

Cianfanelli Francesca Romana *
Bumann Dirk
Biozentrum, Universität Basel , Basel
*Correspondence: Francesca Romana Cianfanelli, f.r.cianfanelli@unibas.ch

Identification of viral transcripts in RNA-seq datasets from bees, ants, wasps and mites

http://jmmcr.microbiologyresearch.org

Brown Katherine *
Olendraite Ingrida
Firth Andrew
University of Cambridge , Cambridge , United Kingdom
*Correspondence: Katherine Brown, kab84@cam.ac.uk

Of mice or men? Developing an ex vivo model of Staphylococcus aureus infection in the cystic fibrosis lung

http://jmmcr.microbiologyresearch.org

Sweeney Esther 1 *
Harrison Freya 1
Guzman Katerina 2
Tormo-Mas M. Angeles 2
1 University of Warwick , Coventry , United Kingdom
2 Instituto de Investigacion Sanitaria La Fe , Valencia , Spain
*Correspondence: Esther Sweeney, e.sweeney@warwick.ac.uk

Inflammation associated ethanolamine facilitates infection by Crohn’s disease-linked adherent-invasive Escherichia coli

http://jmmcr.microbiologyresearch.org

Ormsby Michael 1 *
Logan Michael 2
Johnson Síle 1
McIntosh Anne 1
Hanson Richard 3
Ijaz Umar 2
Russell Richard 3
Gerasimidis Konstantinos 4
Wall Donal 1
1 Institute of Infection, Immunity and Inflammation, University of Glasgow , Glasgow , United Kingdom
2 School of Engineering, University of Glasgow , Glasgow , United Kingdom
3 Department of Paediatric Gastroenterology, Hepatology and Nutrition , Royal Hospital for Children, Glasgow , United Kingdom
4 Human Nutrition, School of Medicine, College of Medical Veterinary and Life Sciences, University of Glasgow , Glasgow , United Kingdom
*Correspondence: Michael Ormsby, michael.ormsby@glasgow.ac.uk

Co-selection of antibiotic resistance caused by a legacy of PTE pollution in Gram-negative bacteria

http://jmmcr.microbiologyresearch.org

Tonner Rebecca 1 *
Peshkur Tanya 1
Rodgers Kiri 2
MacLellan Ian 2
Williams Roderick 2
Hursthouse Andrew 2
Henriquez Fiona 2
Knapp Charles 1
1 University of Strathclyde , Glasgow , United Kingdom
2 University of West of Scotland , Paisley , United Kingdom
*Correspondence: Rebecca Tonner, rebecca.tonner@strath.ac.uk

Use of nanosensor technology to investigate biofilm formation and resulting malodour in washing machines

http://jmmcr.microbiologyresearch.org

Blunk Birte 1 2 *
Perkins Mark 1 2
Walsh Dean 1 2
Chauhan Veeren 2
Camara Miguel 1
Williams Paul 1
Aylott Jonathan 2
Hardie Kim 1
1 Centre for Biomolecular Sciences, University of Nottingham , Nottingham , United Kingdom
2 Boots Science Building, University of Nottingham , Nottingham , United Kingdom
*Correspondence: Birte Hollmann, mbxbh@nottingham.ac.uk

Assembly of a portal-like structure in feline calicivirus following receptor engagement

http://jmmcr.microbiologyresearch.org

Conley Michaela 1 *
McElwee Marion 1
Goodfellow Ian 2
Bhella David 1
1 MRC-University of Glasgow Centre for Virus Research , Glasgow , United Kingdom
2 University of Cambridge , Cambridge , United Kingdom
*Correspondence: Michaela Conley, Michaela.Conley@glasgow.ac.uk

BactiVac, a network to support the study, development and implementation of bacterial vaccines

http://jmmcr.microbiologyresearch.org

Cunningham Adam 1
Dean Johanna 1
Pope Susan 1
Balandyte Evelina 1 *
MacLennan Calman A. 2 3
1 University of Birmingham , Birmingham , United Kingdom
2 University of Oxford , Oxford , United Kingdom
3 Bill and Melinda Gates Foundation , Seattle , USA
*Correspondence: Evelina Balandyte, e.balandyte@bham.ac.uk

Bactericidal fully-human monoclonal antibodies can be cloned from patients convalescing from invasive meningococcal disease

http://jmmcr.microbiologyresearch.org

Bidmos Fadil 1 *
Nadel Simon 1
Screaton Gavin 2
Kroll Simon 1
Langford Paul 1
1 Imperial College London , London , United Kingdom
2 University of Oxford , Oxford , United Kingdom
*Correspondence: Fadil Bidmos, f.bidmos@imperial.ac.uk

The application of CRISPR/Cas9 system in the generation of viral vectored avian influenza vaccines

http://jmmcr.microbiologyresearch.org

Chang Pengxiang *
Sadeyen Jean-Remy
Sealy Joshua
Bhat Sushant
Iqbal Munir
The Pirbright Institute , Woking , United Kingdom
*Correspondence: Pengxiang Chang, pengxiang.chang@pirbright.ac.uk

A novel enterovirus protein modulates infection in gut epithelial cells

http://jmmcr.microbiologyresearch.org

Lulla Valeria 1 *
Dinan Adam 1
Hosmillo Myra 1
Chaudhry Yasmin 1
Sherry Lee 2
Irigoyen Nerea 1
Nayak Komal 1
Stonehouse Nicola 2
Zilbauer Matthias 1
Goodfellow Ian 1
Firth Andrew 1
1 University of Cambridge , Cambridge , United Kingdom
2 University of Leeds , Leeds , United Kingdom
*Correspondence: Valeria Lulla, vl284@cam.ac.uk

Species-specific restriction of Bluetongue virus replication correlates to host resilience

http://jmmcr.microbiologyresearch.org

Hardy Alexandra 1 *
Stewart Meredith 1
Shaw Andrew 1
Varela Mariana 1
Wilson Sam 1
Randall Richard 2
Palmarini Massimo 1
1 MRC-University of Glasgow Centre for Virus Research , Glasgow , United Kingdom
2 University of St Andrews , St Andrews , United Kingdom
*Correspondence: Alexandra Hardy, a.hardy.1@research.gla.ac.uk

Understanding the population dynamics of organisms exposed to the predatory activity of myxobacteria

http://jmmcr.microbiologyresearch.org

Sydney Natashia *
Whitworth David
Aberystwyth University , Aberystwyth , United Kingdom
*Correspondence: Natashia Sydney, nas39@aber.ac.uk

Infectious bronchitis virus modulates cellular stress granule signalling

http://jmmcr.microbiologyresearch.org

Brownsword Matthew 1 2 *
Doyle Nicole 1
Brocard Michele 2
Locker Nicolas 2
Maier Helena 1
1 Pirbright Institute , Woking , United Kingdom
2 University of Surrey , Guildford , United Kingdom
*Correspondence: Matthew Brownsword, matt.brownsword@hotmail.co.uk

Replication of the Chikungunya virus genome requires cellular chloride channels

http://jmmcr.microbiologyresearch.org

Müller Marietta 1 *
Jones Natalie 1
Todd Eleanor 1
Khalid Henna 1
Merits Andres 2
Mankouri Jamel 1
Tuplin Andrew 1
1 University of Leeds , Leeds , United Kingdom
2 University of Tartu , Tartu , Estonia
*Correspondence: Marietta Muller, m.muller@leeds.ac.uk

Host-derived markers of Lyme disease: their discovery and diagnostic potential

http://jmmcr.microbiologyresearch.org

Joyner Greg 1 *
Beeching Nick 1 2
Hiscox Julian 1
Semper Amanda 3
1 University of Liverpool , Liverpool , United Kingdom
2 Liverpool School of Tropical Medicine , Liverpool , United Kingdom
3 Public Health England , Porton Down , United Kingdom
*Correspondence: Greg Joyner, gpjoyner@liverpool.ac.uk

Genome-led discovery of novel microbial natural products

http://jmmcr.microbiologyresearch.org

Russell Alicia *
Lacret Rodney
Truman Andrew
John Innes Centre , Norwich , United Kingdom
*Correspondence: Alicia Russell, alicia.russell@jic.ac.uk

Dimerisation of the influenza virus RNA polymerase during viral genome replication

http://jmmcr.microbiologyresearch.org

Walker Alexander 1 *
Fan Haitian 1
Carrique Loic 2
Keown Jeremy 2
Bauer David 1
Grimes Jonathan 2
Fodor Ervin 1
1 Sir William Dunn School of Pathology , Oxford , United Kingdom
2 Division of Structural Biology , Oxford , United Kingdom
*Correspondence: Alexander Walker, alexander.walker@path.ox.ac.uk

Characterising Birnaviridae replication and reassortment in vitro: virus factories derived from distinct input viruses form in the cytoplasm of co-infected cells and coalesce over time

http://jmmcr.microbiologyresearch.org

Campbell Elle
Wells Joanna
Gray Alice
Broadbent Andrew *
The Pirbright Institute , Woking , United Kingdom
*Correspondence: Andrew Broadbent, andrew.broadbent@pirbright.ac.uk

Encapsidation of viral RNA in Picornavirales: studies on cowpea mosaic virus demonstrate dependence on viral replication

http://jmmcr.microbiologyresearch.org

Peyret Hadrien *
Kruse Inga
Saxena Pooja
Lomonossoff George
John Innes Centre , Norwich , United Kingdom
*Correspondence: Hadrien Peyret, hadrien.peyret@jic.ac.uk

clusterTools: functional element identification for the in silico prioritization of biosynthetic gene clusters

http://jmmcr.microbiologyresearch.org

Lorenzo de los Santos Emmanuel 1 *
Challis Gregory 1 2
1 University of Warwick , Coventry , United Kingdom
2 Monash University , Melbourne , Australia
*Correspondence: Emmanuel Lorenzo de los Santos, e.de-los-santos@warwick.ac.uk

A high throughput siRNA screen to identify host membrane trafficking proteins that restrict pneumovirus egress

http://jmmcr.microbiologyresearch.org

Human Stacey *
Bailey Dalan
Viral Glycoproteins Group, The Pirbright Institute , Woking, Surrey , United Kingdom
*Correspondence: Stacey Human, stacey.human@pirbright.ac.uk

A pilot study to determine the relationship between bacterial populations in the cloaca and the caecum of broiler chickens

http://jmmcr.microbiologyresearch.org

Andreani Nadia *
Goddard Matthew
University of Lincoln , Lincoln , United Kingdom
*Correspondence: Nadia Andreani, nandreani@lincoln.ac.uk

Prevalence of microbial parasites in captive animals across wildlife parks

http://jmmcr.microbiologyresearch.org

Betts Emma 1 *
Gentekaki Eleni 2
Carpenter Angus 3
Harland Adrian 4
Tsaousis Anastasios 1
1 University of Kent , Canterbury , United Kingdom
2 Mae Fah Luang University , Chiang Rai , Thailand
3 Wildwood Trust , Canterbury , United Kingdom
4 Howletts Wild Animal Trust , Canterbury , United Kingdom
*Correspondence: Emma Betts, elb48@kent.ac.uk

Transformation of the dinoflagellate chloroplast

http://jmmcr.microbiologyresearch.org

Howe Christopher *
Nisbet Ellen
Barbrook Adrian
Nimmo Isabel
University of Cambridge , Cambridge , United Kingdom
*Correspondence: Christopher Howe, ch26@cam.ac.uk

Mutasynthesis of novel prodiginines derived from the antibiotic prodigiosin by exploiting substrate specificity of H2MAP oxidases PigB and HapB

http://jmmcr.microbiologyresearch.org

Couturier Maxime *
Bhalara Hiral
Salmond George P.C.
Leeper Finian J.
University of Cambridge , Cambridge , United Kingdom
*Correspondence: Maxime Couturier, mpfc2@cam.ac.uk

Food or a free ride? The ability of a marine microbial community to degrade plastics

http://jmmcr.microbiologyresearch.org

Wright Robyn *
Gibson Matthew
Christie-Oleza Joseph
University of Warwick , Coventry , United Kingdom
*Correspondence: Robyn Wright, R.Wright.1@warwick.ac.uk

Analysis of bacterial competition using imaging mass spectrometry

http://jmmcr.microbiologyresearch.org

Castaño Laia 1 *
Gilmore Ian 2
Johnston Blair 1
Chrubasik Michael 1
Duncan Katherine 1
1 Strathclyde Institue of Pharmacy and Biomedical Sciences, University of Strathclyde , Glasgow , United Kingdom
2 National Physical Laboratory , Teddington , United Kingdom
*Correspondence: Laia Castano, laia.castano-espriu@strath.ac.uk

Identification of sites of Infectious Bronchitis Virus RNA synthesis

http://jmmcr.microbiologyresearch.org

Doyle Nicole *
Rayon Selma
Simpson Jennifer
Hawes Pippa
Maier Helena J
The Pirbright Institute , Pirbright , United Kingdom
*Correspondence: Nicole Doyle, nicole.doyle@pirbright.ac.uk

Evolutionary genomics of the host-restricted pathogen Staphylococcus aureus subsp. anaerobius reveals extensive genome decay and signatures of adaptation

http://jmmcr.microbiologyresearch.org

Yebra Gonzalo 1 *
Neamah Maan M 2
Wee Bryan A 1
Richardson Emily J 1
de la Fuente Ricardo 3
Ross Fitzgerald J 1
Penadés José R 2
1 University of Edinburgh , Edinburgh , United Kingdom
2 University of Glasgow , Glasgow , United Kingdom
3 Universidad Complutense , Madrid , Spain
*Correspondence: Gonzalo Yebra, Gonzalo.Yebra@ed.ac.uk

Understanding how bacterial products from the microbiota enter the host, determining where they aggregate, and their influence over immune cells at these sites

http://jmmcr.microbiologyresearch.org

Jordan Christine *
Clarke Tom
Imperial College London , London , United Kingdom
*Correspondence: Christine Jordan, c.jordan16@imperial.ac.uk

Decaying Ascophyllum nodosum as a source of algal cell wall degrading enzymes with potential utility in enzyme-assisted extraction technologies

http://jmmcr.microbiologyresearch.org

Ihua Maureen 1 *
Mohammed Halimah 1
Guihéneuf Freddy 2
Margassery Lekha 1
Jackson Stephen 1
Clarke David 1
Dobson Alan 1
1 University College Cork , Cork , Ireland
2 National University of Ireland, Galway , Galway , Ireland
*Correspondence: Maureen Ihua, w.ihua@umail.ucc.ie

Development of streptomyces to utilise sustainable feedstock in fermentations

http://jmmcr.microbiologyresearch.org

Birke Anna 1 *
Kendrew Steve 2
Huckle Benjamin 2
Hunter Iain 1
Hoskisson Paul 1
1 University of Strathclyde , Glasgow , United Kingdom
2 GlaxoSmithKline , Worthing , United Kingdom
*Correspondence: Anna Sofia Birke, anna.birke@strath.ac.uk

Mechanistic analysis of the minimalistic twin-arginine translocation system found in Bacillus subtilis

http://jmmcr.microbiologyresearch.org

Cherry Jon *
Palmer Tracy
Newcastle University , Newcastle , United Kingdom
*Correspondence: Jon Cherry, jzcherry@dundee.ac.uk

Generation of recombinant avian coronaviruses indicates the S gene is a factor in pathogenicity

http://jmmcr.microbiologyresearch.org

Stevenson-Leggett Phoebe *
Keep Sarah
Oade Michael
Britton Paul
Bickerton Erica
The Pibright Institute , Woking , United Kingdom
*Correspondence: Phoebe Stevenson-Leggett, phoebe.stevenson-leggett@pirbright.ac.uk

Attenuation of E. coli O157:H7 virulence by a combination of natural plant extracts and organic acids before and after refrigerated storage

http://jmmcr.microbiologyresearch.org

Stratakos Alexandros 1 *
Linton Mark 1
Ward Patrick 2
Sima Filip 1
Kelly Carmel 1
Pinkerton Laurette 1
Gundogdu Ozan 3
Corcionivoschi Nicolae 1
1 Agri-Food and Biosciences Institute , Belfast , United Kingdom
2 Auranta , Dublin , Ireland
3 London School of Hygiene and Tropical Medicine , London , United Kingdom
*Correspondence: Alexandros Stratakos, Alexandros.Stratakos@afbini.gov.uk

Studies of cell surface and soluble HLA class I expression in pancreatic beta cells exposed to interferons or Poly I:C

http://jmmcr.microbiologyresearch.org

Akhbari Pouria *
Morgan Noel
Richardson Sarah
University of Exeter College of Medicine and Health , Exeter , United Kingdom
*Correspondence: Pouria Akhbari, P.Akhbari@exeter.ac.uk

The broad-spectrum antiviral drug arbidol inhibits foot-and-mouth disease virus replication

http://jmmcr.microbiologyresearch.org

Polyak Stephen 1 *
Herod Morgan 2
Adeyemi Oluwapelumi 2
Ward Joseph 2
Tuthill Toby 3
Berryman Stephen 3
Harris Mark 2
Stonehouse Nicola 2
1 University of Washington , Seatte , USA
2 University of Leeds , Leeds , United Kingdom
3 Pirbright Institute , Woking , United Kingdom
*Correspondence: Stephen Polyak, polyak@uw.edu

The effect of Soil pH and phosphorus interactions on nitrous oxide emissions and microbial communities involved in nitrogen cycling

http://jmmcr.microbiologyresearch.org

Grau Butinyac Meritxell 1 2 *
O’Flaherty Vincent 2
Richards Karl 1
Wall David 1
Brennan Fiona 1
1 Teagasc Environmental Research Centre , Wexford , Ireland
2 Microbial Ecology Laboratory, School of Natural Sciences, National University of Ireland Galway , Galway , Ireland
*Correspondence: Meritxell Grau Butinyac, meritxell.grau@teagasc.ie

The microbial diversity of a sulfur-rich and saline cold pool in the Canadian high Arctic

http://jmmcr.microbiologyresearch.org

Christopher Macey Michael 1 *
Stephens Ben 1
Fox-Powell Mark 2
Schwenzer Susanne P. 1
Pearson Victoria K. 1
Cousins Claire R. 2
Olsson-Francis Karen 1
1 The Open University , Milton Keynes , United Kingdom
2 The University of St Andrews , St Andrews , United Kingdom
*Correspondence: Michael Macey, michael.macey@open.ac.uk

Development of in vitro functional antibody assays to identify and assess vaccine targets against E. coli bacteraemia

http://jmmcr.microbiologyresearch.org

Chan Kin *
Hesp Richard
Thomas Steve
Taylor Steve
Leung Stephanie
Penn Elizabeth
Public Health England , Porton Down , United Kingdom
*Correspondence: Kin Chan, kin.chan@phe.gov.uk

Bacterial coping mechanisms for aging: using an individual-based model to study aging in biofilms

http://jmmcr.microbiologyresearch.org

Wright Robyn 1 2 *
Clegg Robert 2
Kreft Jan-Ulrich 2
1 University of Warwick , Coventry , United Kingdom
2 University of Birmingham , Birmingham , United Kingdom
*Correspondence: Robyn Wright, R.Wright.1@warwick.ac.uk

Chain termination of structurally disparate non-ribosomal peptides by a trans-acting β-lactamase

http://jmmcr.microbiologyresearch.org

Fazal Asif 1 *
Thankachan Divya 1
Francis Daniel 1
Song Lijiang 2
Webb Michael 1
Seipke Ryan 1
1 University of Leeds , Leeds , United Kingdom
2 University of Warwick , Coventry , United Kingdom
*Correspondence: Asif Fazal, cm12a2f@leeds.ac.uk

Proteome-wide analysis of CD8+ T cell responses to EBV lytic infection

http://jmmcr.microbiologyresearch.org

Forrest Calum 1 2 *
Hislop Andrew 2
Rickinson Alan 2
Zuo Jianmin 2
1 University College London , London , United Kingdom
2 University of Birmingham , Birmingham , United Kingdom
*Correspondence: Calum Forrest, calum.forrest@ucl.ac.uk

Identification of novel antimicrobial-producing bacteria from an ancient water source by Oxford Nanopore Whole Genome Sequencing and Natural Product Chemistry

http://jmmcr.microbiologyresearch.org

Walker Tim *
Stapleton Paul
Gibbons Simon
University College London , London , United Kingdom
*Correspondence: Tim Walker, tim.walker.14@ucl.ac.uk

Understanding metabolic processes shaping adaptation of E. coli to the gut

http://jmmcr.microbiologyresearch.org

Pedro Miguel 1 *
Batista-Barroso João 2
Pinto Catarina 3
Dias Joana 4
Gordo Isabel 1
Xavier Karina 1
1 IGC – Instituto Gulbenkian de Ciência , Oeiras , Portugal
2 Université de Montréal , Montreal , Canada
3 Faculdade de Ciências , Lisboa , Portugal
4 Instituto de Tecnologia Química e Biológica , Oeiras , Portugal
*Correspondence: Miguel Pedro, mp_miguel_pedro@hotmail.com

Coupling of subunit availability to activation of PMF-driven flagellar type III secretion

http://jmmcr.microbiologyresearch.org

Bryant Owain *
Chung Betty
Fraser Gillian
University of Cambridge , Cambridge , United Kingdom
*Correspondence: Owain Bryant, ojb29@cam.ac.uk

Genome epidemiology of Mycobacterium bovis infection in contemporaneous, sympatric badger and cattle populations in Northern Ireland

http://jmmcr.microbiologyresearch.org

Allen Adrian 1 *
McAdam Paul 1
Presho Eleanor 1
Hughes Carolyn 1
Rutherford David 1
Bell Colin 1
Watson Lucy 1
Mullan Grainne 1
Kao Rowland 2
Crispell Joseph 3
Akhmetova Assel 4
Menzies Fraser 5
McCormick Carl 5
McGuckian Paddy 5
Kirke Raymond 5
Skuce Robin 1 6
1 Agri-Food and Biosciences Institute , Belfast , United Kingdom
2 University of Edinburgh , Edinburgh , United Kingdom
3 University College Dublin , Dublin , Ireland
4 University of Glasgow , Glasgow , United Kingdom
5 Department of Agriculture, Environment and Rural Affairs , Belfast , United Kingdom
6 Queen’s university Belfast , Belfast , United Kingdom
*Correspondence: Adrian Allen, adrian.allen@afbini.gov.uk

An uncharacterised protein mediates motility, biofilm formation, and host colonisation in adherent invasive Escherichia coli

http://jmmcr.microbiologyresearch.org

Cogger-Ward Robert *
Collins Adam
Dehinsilu Jacob
Huett Alan
University of Nottingham , Nottingham , United Kingdom
*Correspondence: Robert Cogger-Ward, robert.cogger-ward@nottingham.ac.uk

Genomic analysis of Burkholderia ambifaria identifies key specialised metabolites for biopesticidal applications

http://jmmcr.microbiologyresearch.org

Mullins Alex 1 *
Murray James 1
Bull Matthew 1
Jenner Matthew 2
Jones Cerith 1 3
Webster Gordon 1
Green Angharad 4
Neill Daniel 4
Connor Thomas 1
Parkhill Julian 5
Challis Gregory 2 6
Mahenthiralingam Eshwar 1
1 Cardiff University , Cardiff , United Kingdom
2 University of Warwick , Coventry , United Kingdom
3 University of South Wales , Cardiff , United Kingdom
4 University of Liverpool , Liverpool , United Kingdom
5 Wellcome Sanger Institute , Cambridge , United Kingdom
6 Monash University , Clayton , Australia
*Correspondence: Alex Mullins, mullinsa@cardiff.ac.uk

Development of low bio-containment assays to characterise the antibody responses in pigs to Nipah virus vaccine candidates

http://jmmcr.microbiologyresearch.org

Thakur Nazia 1 *
Nath Barman Nagendra 2
Chang Li-Yen 3
Chappell Keith 4
Gilbert Sarah 5
Lambe Teresa 5
Marsh Glenn 6
McLean Rebecca 1
Mourino Mercedes 7
Pedrera Miriam 1
Raue Rudiger 9
Tchilian Elma 1
Watterson Daniel 4
Young Paul 4
Graham Simon 1
Bailey Dalan 1
1 The Pirbright Institute , Woking , United Kingdom
2 Assam Agricultural University , Assam , India
3 University of Malaya , Kuala Lumpur , Malaysia
4 University of Queensland , Brisbane , Australia
5 Jenner Institute, University of Oxford , Oxford , United Kingdom
6 CSIRO Health and Biosecurity, Australian Animal Health Laboratory , Geelong , Australia
7 Zoetis, Ctra , Camprodon , Spain
8 The Pirbright Institute , Woking , United States Minor Outlying Islands
9 Zoetis , Zaventem , Belgium
10 University of Queensland , Brisbane , Azerbaijan
*Correspondence: Nazia Thakur, nazia.thakur@pirbright.ac.uk

Development of serologic tools for diagnosis and surveillance of Chikungunya

http://jmmcr.microbiologyresearch.org

Fonseca Bagno Flávia 1 2 *
Marta Figueiredo Maria 2
Carvalho Godoi Lara 2
Guimarães da Fonseca Flávio 2 1
1 Universidade Federal de Minas Gerais , Belo Horizonte , Brazil
2 Centro de Tecnologia de Vacinas , Belo Horizonte , Brazil
*Correspondence: Flavia Bagno, flavia.bagno@gmail.com

HSV-1 genome decompaction plays an important role in viral DNA sensing by PML-NB intrinsic antiviral regulators

http://jmmcr.microbiologyresearch.org

Iliev Victor *
Orr Anne
Boutell Chris
Centre for Virus Research – University of Glasgow , Glasgow , United Kingdom
*Correspondence: Victor Iliev, v.iliev179@gmail.com

The macrophage intracellular niche and its role in cryptococcosis

http://jmmcr.microbiologyresearch.org

Pline Katherine *
Johnston Simon
Bradford James
University of Sheffield , Sheffield , United Kingdom
*Correspondence: Katherine Pline, kmpline1@sheffield.ac.uk

The role of RNA-RNA interactions in the assembly and reassortment of influenza A viruses

http://jmmcr.microbiologyresearch.org

Knight Michael 1 *
Dadonaite Bernadeta 1
Gilbertson Brad 2
Trifkovic Sanja 2
Rockman Steven 2
Laederach Alain 3
Brown Lorena 2
Fodor Ervin 1
Bauer David LV 1
1 Sir William Dunn School of Pathology, University of Oxford , Oxford , United Kingdom
2 Department of Microbiology and Immunology, The University of Melbourne , Melbourne , Australia
3 Department of Biology, University of North Carolina , Chapel Hill , USA
*Correspondence: Michael Knight, michael.knight@path.ox.ac.uk

In silico identification of two novel antimicrobial peptides with antibacterial activity against multi-drug resistant Staphylococcus aureus

http://jmmcr.microbiologyresearch.org

Oyama Linda 1 *
Olleik Hamza 2
Carolina Nery Teixeira Ana 3
Guidini Matheus M 3
Pickup James A 1
Cookson Alan R 4
Vallin Hannah 4
Wilkinson Toby 5
Bazzolli Denise 3
Richards Jennifer 6
Wootton Mandy 6
Mikut Ralf 7
Hilpert Kai 8
Maresca Marc 2
Perrier Josette 9
Hess Matthias 10
Mantovani Hilario C 11
Fernandez-Fuentes Narcis 4
Creevey Christopher J 1
Huws Sharon A 1
1 Queen’s University Belfast , Belfast , United Kingdom
2 Aix Marseille University , Marseille , France
3 Universidade Federal de Viçosa , Viçosa , Brazil
4 Aberystwyth University , Aberystwyth , United Kingdom
5 University of Edinburgh , Edinburgh , United Kingdom
6 Public Health Wales NHS , Cardiff , United Kingdom
7 Karlsruhe Institute of Technology , Leopoldshafen , Germany
8 St. George’s University of London , London , United Kingdom
9 Aix Marseille University , Marseille , France
10 UC Davis, College of Agricultural and Environmental Sciences , California , USA
11 Unversidade Federal de Viçosa , Viçosa , Brazil
*Correspondence: Linda Oyama, l.oyama@qub.ac.uk

In vivo and ex vivo models of infectious bursal disease virus (IBDV) in inbred chicken lines differing in their resistance to the disease

http://jmmcr.microbiologyresearch.org

Asfor Amin 1 *
Dulwich Kate 2
Broadbent Andrew 2
1 The Pirbright institute , Woking, Surrey , United Kingdom
2 The Pirbright institute , Woking, Surrey Gu24 0NF , United Kingdom
*Correspondence: Amin Asfor, amin.asfor@pirbright.ac.uk

Global mapping of protein subcellular location in apicomplexans: the parasite as we’ve never seen it before

http://jmmcr.microbiologyresearch.org

Barylyuk Konstantin 1
Koreny Ludek 1
Ke Huiling 1
Butterworth Simon 1
Lassadi Imen 1
Mourier Tobias 2
Breckels Lisa 1
Gatto Laurent 1
Pain Arnab 2
Lilley Kathryn 1
Waller Ross 1 *
1 University of Cambridge , Cambridge , United Kingdom
2 King Abdullah University of Science and Technology , Jeddah , Saudi Arabia
*Correspondence: Ross Waller, rfw26@cam.ac.uk

Human Cytomegalovirus pUL83 targets core histones to inhibit interferon synthesis and promote viral spread

http://jmmcr.microbiologyresearch.org

Deb Anasua *
Paulus Christina
Nevels Michael
University of St Andrews , St Andrews , United Kingdom
*Correspondence: Anasua Deb, ad279@st-andrews.ac.uk

Mapping the pH sensors critical for host cell entry by a complex Nonenveloped Virus

http://jmmcr.microbiologyresearch.org

Wu Weining *
Roy Polly
LSHTM , London , United Kingdom
*Correspondence: Weining Wu, bellhust@hotmail.com

The great escape: dissecting the interactions between Mycobacterium bovis and the soil amoeba Dictyostelium discoideum

http://jmmcr.microbiologyresearch.org

Butler Rachel 1 *
Smith Alex 1
Mendum Tom 1
Chandran Aneesh 1
Wu Huihai 1
Lefrancois Louise 2
Soldati Thierry 2
Stewart Graham 1
1 University of Surrey , Guildford , United Kingdom
2 University of Geneva , Geneva , Switzerland
*Correspondence: Rachel Butler, r.shrimpton@surrey.ac.uk

Investigation of the Candida-host interaction using dual RNA-seq

http://jmmcr.microbiologyresearch.org

Iracane Elise 1 *
Hovhannisyan Hrant 2
Miguel Oliveira Pacheco João 1
Pekmezovic Marina 3
Hube Bernhard 3 4 5
Gabaldón Toni 2 6 7
Butler Geraldine 1
1 University College Dublin , Dublin , Ireland
2 Centre for Genomic Regulation, The Barcelona Institute of Science and Technology , Barcelona , Spain
3 Department of Microbial Pathogenicity Mechanisms, Hans Knöll Institute , Jena , Germany
4 Friedrich-Schiller University , Jena , Germany
5 Center for Sepsis Control and Care, Jena University Hospital , Jena , Germany
6 Universitat Pompeu Fabra , Barcelona , Spain
7 Institució Catalana de Recerca i Estudis Avançats , Barcelona , Spain
*Correspondence: Elise Iracane, elise.iracane@ucd.ie

Modulation of arbovirus infection by mosquito saliva

http://jmmcr.microbiologyresearch.org

Lefteri Daniella 1 *
Pondeville Emilie 2
Bryden Steven 2
Pingen Marieke 2
McKimmie Clive 1
1 University of Leeds , Leeds , United Kingdom
2 University of Glasgow , Glasgow , United Kingdom
*Correspondence: Daniella Lefteri, bs11dl@leeds.ac.uk

Investigating the mechanisms of BK polyomavirus egress and virus-host interactions

http://jmmcr.microbiologyresearch.org

Ho Sophia *
Caller Laura
Beale Rupert
Crump Colin
University of Cambridge , Cambridge , United Kingdom
*Correspondence: Sophia Ho, sh0437@my.bristol.ac.uk

Metagenomic analysis of open-air and indoor spent fuel storage ponds at Sellafield, UK

http://jmmcr.microbiologyresearch.org

Ruiz Lopez Sharon 1 *
Foster Lynn 2
Song Hokyung 3
Cole Nick 2
Adams Jonathan 4
Lloyd Jonathan 1
1 University of Manchester , Manchester , United Kingdom
2 Sellafield Ltd , Cumbria , United Kingdom
3 Seoul National University , Seoul , Korea Republic of
4 Cranfield University , Bedford , United Kingdom
*Correspondence: Sharon Ruiz Lopez, sharulo_268@hotmail.com

Type I interferon activity promotes a cellular environment that supports the establishment of latency by human cytomegalovirus

http://jmmcr.microbiologyresearch.org

Olaizola-Rebe Paula 1
Onyerindu Chinedu 1
Kiesworo Kevin 1
Lodha Manivel 1
Stamminger Thomas 2
Peters Nicholas 1
Murray Matthew 1 *
Reeves Matthew 1
1 University College London , London , United Kingdom
2 Friedrich-Alexander University , Erlangen-Nuremberg , Germany
*Correspondence: Matthew Murray, matthew.murray@ucl.ac.uk

Investigating the impact of Herpes simplex virus type 1 latency-associated non-coding RNAs on apoptosis in human neuronal cells

http://jmmcr.microbiologyresearch.org

Jacobs Amy 1 2 *
Efstathiou Stacey 3
Nicoll Michael 1
1 The National Institute for Biological Standards and Control , Potters Bar , United Kingdom
2 Imperial College London , London , United Kingdom
3 University of Cambridge , Cambridge , United Kingdom
*Correspondence: Amy Jacobs, amy.jacobs@nibsc.org

Enhancing the unexplored chemical diversity of Streptomyces sp. to produce new antibiotics active against multidrug resistant Acinetobacter baumannii

http://jmmcr.microbiologyresearch.org

Burns Joshua 1 2 *
Law Samantha 2
Edwards Christine 1
Lawton Linda 1
1 Robert Gordon University , Aberdeen , United Kingdom
2 NCIMB Ltd , Aberdeen , United Kingdom
*Correspondence: Joshua Burns, j.burns5@rgu.ac.uk

Investigating the role of the bacterial mechanosensitive channel YnaI in Salmonella pathogenesis

http://jmmcr.microbiologyresearch.org

Asogwa Mimi 1 *
Miller Samantha 1
Spano Stefania 1
Stevens Mark 2
1 University of Aberdeen , Aberdeen , United Kingdom
2 University of Edinburgh , Edinburgh , United Kingdom
*Correspondence: Mimi Asogwa, r01mna16@abdn.ac.uk

Characterization the role of key DNA sensors in herpes simplex virus replication

http://jmmcr.microbiologyresearch.org

Moshrif Hanan 1 2 *
O’Hare Peter 1
1 Imperial College London , London , United Kingdom
2 Taibah University , Madinah , Saudi Arabia
*Correspondence: Hanan Moshrif, h.moshrif16@imperial.ac.uk

Global gene expression profiling of a virulent Klebsiella pneumoniae strain during pulmonary infection

http://jmmcr.microbiologyresearch.org

Kidd Timothy 1 2 *
Dumigan Amy 2
Forde Brian 1
Phan Minh-Duy 1
Canals Rocio 3
Hinton Jay 3
Schembri Mark 1
Bengoechea Jose 2
1 School of Chemistry and Molecular Biosciences, The University of Queensland , Brisbane , Australia
2 Wellcome-Wolfson Institute of Experimental Medicine, Queen’s University Belfast , Belfast , United Kingdom
3 Institute of Integrative Biology, University of Liverpool , Liverpool , United Kingdom
*Correspondence: Timothy Kidd, t.m.kidd@uq.edu.au

Genomic evolution of Klebsiella pneumoniae clones: the good, the bad and the ugly

http://jmmcr.microbiologyresearch.org

Wyres Kelly 1 *
Wick Ryan 1
Judd Louise 1
Froumine Roni 1
Tokolyi Alex 1
Gorrie Claire 1
Lam Margaret 1
Duchêne Sebastián 1
Jenney Adam 2
Holt Kathryn 1 3
1 University of Melbourne , Parkville , Australia
2 The Alfred Hospital , Melbourne , Australia
3 London School of Hygiene and Tropical Medicine , London , United Kingdom
*Correspondence: Kelly Wyres, kelly.wyres@googlemail.com

Why does a heterotrophic marine protist produce carotenoids? Genetic approaches to investigate the ecophysiology of the thraustochytrid Aurantiochytrium limacinum

http://jmmcr.microbiologyresearch.org

Rius Mariana
Rest Joshua
Collier Jackie *
Stony Brook University , Stony Brook , USA
*Correspondence: Jackie Collier, jackie.collier@stonybrook.edu

The genomic (re)definition of EPEC

http://jmmcr.microbiologyresearch.org

Heinz Eva 1 2 *
Mather Alison 3 2
Petty Nicola 4 5
Kallonen Teemu 6 5
Semmler Torsten 7
Wieler Lothar 7
Elias Waldir 8
Regua-Mangia Adriana 9
Corander Jukka 6 5
Gomez Tânia 10
Jenkins Claire 11
Frankel Gad 12
Thomson Nicholas 5 13
1 Liverpool School of Tropical Medicine , Liverpool , United Kingdom
2 Wellcome Trust Sanger Centre , Hinxton , United Kingdom
3 Quadrate Institute Bioscience , Norwich , United Kingdom
4 University of Technology Sydney , Sydney , Australia
5 Wellcome Trust Sanger Institute , Hinxton , United Kingdom
6 University of Oslo , Oslo , Norway
7 Robert Koch Institute , Berlin , Germany
8 Instituto Butantan , São Paulo , Brazil
9 Escola Nacional de Saúde Pública Sergio Arouca , Fundação Oswaldo Cruz, Rio de Janeiro , Brazil
10 Universidade Federal de São Paulo , São Paulo , Brazil
11 Public Health England , London , United Kingdom
12 Imperial College London , London , United Kingdom
13 London School of Hygiene and Tropical Medicine , London , United Kingdom
*Correspondence: Eva Heinz, eva.heinz@lstmed.ac.uk

Uncovering the molecular basis of viable but non culturable (VBNC) cells

http://jmmcr.microbiologyresearch.org

Wagley Sariqa *
Titball Richard
Butler Clive
Univerisity of Exeter , Exeter , United Kingdom
*Correspondence: Sariqa Wagley, s.wagley@exeter.ac.uk

Developing genetic manipulation platforms for Naegleria gruberi

http://jmmcr.microbiologyresearch.org

Kazana Eleanna
von der Haar Tobias
Tsaousis Anastasios D. *
University of Kent, School of Biosciences , Canterbury , United Kingdom
*Correspondence: Anastasios Tsaousis, a.tsaousis@kent.ac.uk

Clinical management of low-level hepatitis B surface antigen in haemodialysis patients with a recent history of HBV vaccination – the results of a UK-wide survey

http://jmmcr.microbiologyresearch.org

Goddard Megan *
Timms Judith
Virology and Molecular Pathology Department, University Hospitals Coventry and Warwickshire NHS Trust , Coventry , United Kingdom
*Correspondence: Megan Goddard, megan.goddard@uhcw.nhs.uk

A large-scale investigation of stress response mechanisms in the industrial yeast Kluyveromyces marxianus

http://jmmcr.microbiologyresearch.org

Montini Noemi 1 *
Doughty Tyler 2
Dmenzain Del Castillo Cerecer Ivan 2
Varela Javier 1
Fenton Darren Anthony 1
Baranov Pavel 2
Nielsen Jens 2
Morrissey John 1
1 University College Cork , Cork , Ireland
2 Chalmers University of Technology , Gothenburg , Sweden
Siewers Verena 2
*Correspondence: Noemi Montini, n.montini92@gmail.com

Effects of an RNA chaperone on mutation tolerance

http://jmmcr.microbiologyresearch.org

Soo Valerie *
Warnecke Tobias
London Institute of Medical Sciences , London , United Kingdom
*Correspondence: Valerie Soo, v.soo@lms.mrc.ac.uk

The biocide triclosan triggers multiple regulatory systems in Staphylococcus aureus to induce antibiotic tolerance

http://jmmcr.microbiologyresearch.org

Walsh Dean *
Aylott Jonathan
Hardie Kim
University of Nottingham , Nottingham , United Kingdom
*Correspondence: Dean Walsh, stxdw4@nottingham.ac.uk

Using quantitative proteomics to analyse HCMV manipulation of dendritic cells following cell-cell transfer

http://jmmcr.microbiologyresearch.org

Kerr Lauren 1 *
Soday Lior 2
Weekes Michael 2
Von Ruhland Chris 1
Wang Edward 1
Stanton Richard 1
1 Cardiff University , Cardiff , United Kingdom
2 Cambridge University , Cambridge , United Kingdom
*Correspondence: Lauren Kerr, kerrle@cardiff.ac.uk

Multi-scale variability analysis of Arctic soil microbial communities

http://jmmcr.microbiologyresearch.org

Malard Lucie *
Pearce David
Northumbria University , Newcastle upon Tyne , United Kingdom
*Correspondence: Lucie Malard, lucie.malard@northumbria.ac.uk

Flavivirus membrane (M) proteins as potential ion channel antiviral targets

http://jmmcr.microbiologyresearch.org

Brown Emma *
Beaumont Hannah
Lefteri Daniella
Bentham Matthew
Foster Richard
McKimmie Clive
Kalli Antreas
Griffin Stephen
Leeds University , Leeds , United Kingdom
*Correspondence: Emma Brown, bs13etb@leeds.ac.uk

The ‘missing’ gastric microbe; the impact of gastric cancer-associated microbiota on Helicobacter pylori growth in vitro and its implications in gastric carcinogenesis

http://jmmcr.microbiologyresearch.org

Alfawaz Dana 1
Katsande Paidamoyo 1
Jordan Helen 1
Kuehne Sarah 1
Ferrero Richard 2
Rossiter Amanda 1 *
1 University of Birmingham , Birmingham , United Kingdom
2 Hudson Institute of Medical Research , Melbourne , Australia
*Correspondence: Amanda Rossiter, rossiterae@gmail.com

Proteomic analysis reveals vector-virus interactions between Zika virus and Aedes aegypti mosquito cells

http://jmmcr.microbiologyresearch.org

Gestuveo Rommel 1 2 *
Varjak Margus 1
Kohl Alain 1
1 MRC-University of Glasgow Centre for Virus Research , Glasgow , United Kingdom
2 University of the Philippines Visayas , Iloilo , Philippines
*Correspondence: Rommel Gestuveo, r.gestuveo.1@research.gla.ac.uk

Mastitis and animal husbandry – high-throughput sequencing as a support tool

http://jmmcr.microbiologyresearch.org

Blake Daniel 1 2 *
Haynes Edward 1
Kyriazakis Ilias 2
1 FERA Science Limited , York , United Kingdom
2 Newcastle University , Newcastle , United Kingdom
*Correspondence: Daniel Blake, daniel.blake@fera.co.uk

Unravelling a co-nsP-iracy: the role of chikungunya virus non-structural protein 3 in replication and pathogenesis

http://jmmcr.microbiologyresearch.org

Lee Siu Yi *
Stonehouse Nicola
Harris Mark
University of Leeds , Leeds , United Kingdom
*Correspondence: Siu Yi Lee, siuyilee95@gmail.com

From Antarctic DNA to stress tolerant crop plants – exploiting the why protein domain

http://jmmcr.microbiologyresearch.org

Mertens Jasmin 1 *
Anderson Dominique 2
Ferreras Eloy 1
Cowan Don 1
1 Centre for Microbial Ecology and Genomics, University of Pretoria , Pretoria , South Africa
2 Institute for Microbial Biotechnology and Metagenomics, University of the Western Cape , Cape Town , South Africa
*Correspondence: Jasmin Mertens, jasmin.mertens@up.ac.za

Characterisation of bovine dendritic cells following FMDV infection

http://jmmcr.microbiologyresearch.org

Yasmin Amina 1 2 *
Moffat Katy 1
Reid Elizabeth 1
Charleston Bryan 1
Milling Simon 2
Seago Julian 1
1 The Pirbright Institute , Woking , United Kingdom
2 Glasgow University , Glasgow , United Kingdom
*Correspondence: amina yasmin, amina.yasmin@pirbright.ac.uk

Using a metabolic model of Acetobacterium woodii for insights into its utility for biotechnological purposes

http://jmmcr.microbiologyresearch.org

Mesfin Noah *
Fell David
Mesfin Noah *
Oxford Brookes University , Oxford , United Kingdom
*Correspondence: Noah Mesfin, n.mesfin@brookes.ac.uk

Phylogenetics and vector competence of a bovine ephemeral fever virus strain from Israel

http://jmmcr.microbiologyresearch.org

Fernandez de Marco Mar 1
Hernandez-Triana Luis 1
Dorey-Robinson Daniel 1
McElhinney Lorraine 1
Sanders Chris 2
Carpenter Simon 2
Fooks Anthony 1
Zalensky Olga 3
Gelman Boris 3
Erster Oran 3
Johnson Nicholas 1 *
1 Animal and Plant Health Agency , Addlestone , United Kingdom
2 The Pirbright Institute , Pirbright , United Kingdom
3 The Kimron Institute , Bet Dagon , Israel
*Correspondence: Nicholas Johnson, Nick.Johnson@apha.gov.uk

Characterisation of new environmental bacteriophages targeting the Escherichia coli LamB outer membrane porin

http://jmmcr.microbiologyresearch.org

Zeng Ziyue *
University of Cambridge , Cambridge , United Kingdom
*Correspondence: Ziyue Zeng, hlzzeng@gmail.com

The role of SARM in the control of immune response driven by Klebsiella pneumonia infection

http://jmmcr.microbiologyresearch.org

Feriotti Claudia *
Bengoechea Jose
Queen’s University , Belfast , United Kingdom
*Correspondence: Claudia Feriotti, c.feriotti@qub.ac.uk

Evolutionary strategies of Bdellovibrio bacteriovorus predators and prey

http://jmmcr.microbiologyresearch.org

Summers Kim *
Kreft Jan-Ulrich
University of Birmingham , Birmingham , United Kingdom
*Correspondence: Kimberley Summers, jks282@bham.ac.uk

Generation, lyophilisation and epitope modification of high titre filovirus pseudotyped lentiviruses for use in antibody neutralisation assays and ELISA

http://jmmcr.microbiologyresearch.org

Mayora-Neto Martin 1 *
Bentley Emma 2
Wright Edward 3
Temperton Nigel 1
Ploemen Ivo 4
Soema Peter 4
ten Have Rimko 4
Masterson Stuart 5
Michaelis Martin 5
Wass Mark 5
Scott Simon 1
1 University of Kent , Chatham , United Kingdom
2 National Institute for Biological Standards and Control , Potters Bar , United Kingdom
3 University of Sussex , Brighton , United Kingdom
4 Intravacc , Bilthoven , Netherlands
5 University of Kent , Canterbury , United Kingdom
*Correspondence: Martin Mayora Neto, mm930@kent.ac.uk

Identification of putative packaging signals in the RNA of foot-and-mouth disease virus (FMDV)

http://jmmcr.microbiologyresearch.org

Neil Chris 1 *
Logan Grace 1
Newman Joseph 1
Ward Joseph 2
Rowlands David 2
Stonehouse Nic 2
Tuthill Toby 1
1 The Pirbright Institute , Pirbright , United Kingdom
2 University of Leeds , Leeds , United Kingdom
*Correspondence: Chris Neil, chris.neil@pirbright.ac.uk

Discovering the biology behind the organism while developing genetic tools for Corallochytrium limacisporum

http://jmmcr.microbiologyresearch.org

Kozyczkowska Aleksandra
Najle Sebastian
Ruiz-Trillo Iñaki
Casacuberta Elena *
Institute of Evolutionary Biology , IBE (CSIC-University Pompeu Fabra, Barcelona , Spain
*Correspondence: Elena Casacuberta, elena.casacuberta@ibe.upf-csic.es

The African horse sickness virus NS4 counteracts the antiviral response and is a determinant of viral virulence

http://jmmcr.microbiologyresearch.org

Jin Yi 1
Bakshi Siddharth 1
Caporale Marco 2
Taggart Aislynn 1
Stewart Meredith 1 *
Palmarini Massimo 1
1 MRC-University of Glasgow Centre for Virus Research , Glasgow , United Kingdom
2 ISZ Istituto Zooprofilaticco Spermentale , Teramo , Italy
*Correspondence: Meredith Stewart, Meredith.Stewart@glasgow.ac.uk

The co-occurrence and co-exclusion of evolving objects in prokaryotes

http://jmmcr.microbiologyresearch.org

Whelan Fiona J 1 *
Rusilowicz Martin 2
McInerney James O 1
1 University of Nottingham , Nottingham , United Kingdom
2 University of Manchester , Manchester , United Kingdom
*Correspondence: Fiona J Whelan, fiona.whelan@nottingham.ac.uk

Human papillomavirus E6 regulates the trafficking of gap junction protein Cx43

http://jmmcr.microbiologyresearch.org

Dong Li 1 *
Johnstone Scott 2
Graham Sheila 1
1 MRC-central for Virus research, University of Glasgow , Glasgow , United Kingdom
2 University of Virginia School of Medicine, Robert M Berne Cardiovascular Research Centre , Virginia , USA
*Correspondence: Li Dong, l.dong.1@research.gla.ac.uk

Cross-sectional study of respiratory Aspergillus spp. colonization or infection in patients with various stages of chronic obstructive pulmonary disease (COPD) using culture vs non-culture based technique

http://jmmcr.microbiologyresearch.org

Waqas Sarmad 1 2 *
Dunne Katie 1
Talento Alida Fe 2
Wilson Graham 2
Martin-Loeches Ignacio 2
Keane Joseph 1 2
Rogers Thomas R 1 2
1 Trinity College Dublin , Dublin , Ireland
2 St. James’s Hospital , Dublin , Ireland
*Correspondence: Sarmad Waqas, sarmadw234@gmail.com

Modification of the ADP-ribose-1"-monophosphatase domain in recombinant infectious bronchitis virus affects viral replication in vitro and attenuates the virus in vivo

http://jmmcr.microbiologyresearch.org

Dowgier Giulia *
Keep Sarah
Britton Paul
Bickerton Erica
The Pirbright Institute, Pirbright , Woking , United Kingdom
*Correspondence: Giulia Dowgier, giulia.dowgier@pirbright.ac.uk

Marine Streptomyces spp. isolates with synthetic polyesters-degrading activity

http://jmmcr.microbiologyresearch.org

Leao de Almeida Eduardo 1 *
Felipe Carrillo Rincón Andrés 1
Nevalainen Seija Elizabeth 1
Jackson Stephen 1
O’Leary Niall 1 2
Dobson Alan 1 2
1 School of Microbiology, University College Cork , Cork , Ireland
2 Environmental Research Institute, University College Cork , Cork , Ireland
*Correspondence: Eduardo Leao de Almeida, e.leaodealmeida@umail.ucc.ie

Microbiology in primary school teaching

http://jmmcr.microbiologyresearch.org

Crossman Lisa 1 2 *
1 SequenceAnalysis.co.uk , Norwich , United Kingdom
2 School of Biological Sciences, University of East Anglia , Norwich , United Kingdom
*Correspondence: Lisa Crossman, l.crossman@uea.ac.uk

SNP based transmission study of badgers infected with Mycobacterium bovis in the edge risk area of England

http://jmmcr.microbiologyresearch.org

O’Cathail Colman 1 *
Barron Elsa Sandoval 1
Swift Ben 2
Bennett Malcolm 1
Emes Richard 1
1 University of Nottingham , Nottingham , United Kingdom
2 Royal Veterinary College , London , United Kingdom
*Correspondence: Colman O’Cathail, colman.o’cathail@nottingham.ac.uk

The persistence and dynamics of commensal poultry gut flora within a broiler rearing house

http://jmmcr.microbiologyresearch.org

Brown Helen 1 *
Healy Sara 1
Pursley Isabella 1
Parker Daniel 2
Hayes Grant 2
Horton Daniel 1
Ragione Roberto La 1
Neilson David 3
1 University of Surrey , Guildford , United Kingdom
2 Slate Hall Veterinary Practice , Metheringham , United Kingdom
3 Avara Foods Ltd , Brackley , United Kingdom
*Correspondence: Helen Brown, helenlouise.brown@surrey.ac.uk

Of microscopes and microbes; novel applications of optical microscopy to microbiology

http://jmmcr.microbiologyresearch.org

Rooney Liam *
Hoskisson Paul
McConnell Gail
University of Strathclyde , Glasgow , United Kingdom
*Correspondence: Liam Rooney, liam.rooney@strath.ac.uk

Role of BPIFA1 in the pathogenesis and immune response against Influenza A virus in mice

http://jmmcr.microbiologyresearch.org

Al Katy Sanaria 1 2 *
Kipar Anja 3
Stewart James 1
1 Department of Infection Biology, Institute of Infection and Global Health, University of Liverpool , Liverpool , United Kingdom
2 Department of Pathology and Poultry Diseases, College of Veterinary Medicine, University of Mosul , Mosul , Iraq
3 Institute of Veterinary Pathology, University of Zurich , Zurich , Switzerland
*Correspondence: Sanaria Al Katy, s.h.m.al-katy@liverpool.ac.uk

PorA-Loop4 derived peptides of Neisseria meningitidis cause a G1 cell cycle arrest through the Akt signalling pathway in human brain microvascular endothelial cells

http://jmmcr.microbiologyresearch.org

Firdaus Rininta 1 2 *
Oldfield Neil J. 1
Wooldridge Karl G. 1
1 University of Nottingham , Nottingham , United Kingdom
2 Faculty of Pharmacy, Pancasila University , Jakarta Selatan , Indonesia
*Correspondence: Rininta Firdaus, msxrf3@nottingham.ac.uk

Discovery of novel highly divergent RNA viruses in European rodents and rabbits

http://jmmcr.microbiologyresearch.org

Tsoleridis Theocharis 1 *
Chappell Joseph 1
Onianwa Okechukwu 1
Monchatre-Leroy Elodie 2
Umhang Gérald 2
Shi Mang 3
Tarlinton Rachael 4
McClure Patrick 1
Holmes Edward 3
Ball Jonathan 1
1 School of Life Sciences, University of Nottingham , Nottingham , United Kingdom
2 Anses, Laboratoire de la rage et de la faune sauvage , Malzéville , France
3 Marie Bashir Institute for Infectious Diseases and Biosecurity, Charles Perkins Centre, School of Life and Environmental Sciences and Sydney Medical School, The University of Sydney , Sydney , Australia
4 School of Veterinary Medicine and Science, University of Nottingham, Sutton Bonington Campus , Loughborough , United Kingdom
*Correspondence: Theocharis Tsoleridis, t.tsoleridis@nottingham.ac.uk

Waste not, want not: enhancing the ability of yeast to utilise its own leftovers from the brewing industry to fuel the transport industry with ethanol

http://jmmcr.microbiologyresearch.org

Beaton Ainsley 1 *
Tucker Nicholas 1
Escalettes Franck 2
Magneschi Leonardo 2
1 University of Strathclyde , Glasgow , United Kingdom
2 Ingenza Ltd , Edinburgh , United Kingdom
*Correspondence: Ainsley Beaton, ainsley.beaton@strath.ac.uk

Repurposing histone deacetylase inhibitors (HDACi) to treat Candida glabrata infections

http://jmmcr.microbiologyresearch.org

O’Kane Callum J. 1
Marshall Andrew 1
O’Connell Mary J. 2
Hyland Edel M. 1 *
1 Queens University Belfast , Belfast , United Kingdom
2 Nottingham University , Nottingham , United Kingdom
*Correspondence: Edel Hyland, e.hyland@qub.ac.uk

Influence of gestational and developmental age on human airway epithelial innate immune responses to Respiratory Syncytial Virus (RSV) in early life

http://jmmcr.microbiologyresearch.org

Groves Helen *
Broadbent Lindsay
Guo-Parke Hong
Shields Mike
Power Ultan
Queens University Belfast , Belfast , United Kingdom
*Correspondence: Helen Groves, hgroves01@qub.ac.uk

St. Abb’s Head phlebovirus – a separate virus species or a strain of Uukuniemi phlebovirus?

http://jmmcr.microbiologyresearch.org

Szemiel Agnieszka M *
Willett Brian J
Centre for Virus Research, University of Glasgow , Glasgow , United Kingdom
*Correspondence: Agnieszka Szemiel, agnieszka.szemiel@glasgow.ac.uk

Modified time-temperature combinations reduce beef carcass contamination but will this benefit be seen thoughout the food chain?

http://jmmcr.microbiologyresearch.org

Koolman Leonard 1 *
McSharry Siobhan 1 2
Whyte Paul 2
Bolton Declan 1
1 Teagasc Food Research Centre , Dublin , Ireland
2 University College Dublin , Dublin , Ireland
*Correspondence: Leonard Koolman, leonard.koolman@teagasc.ie

Transcriptomic analysis indicates the mode of action of the novel antibiotic MGB-BP-3 against Staphylococcus aureus

http://jmmcr.microbiologyresearch.org

Nieminen Leena 1
Lemonidis Kimon 2
Browning Douglas 3
Hunter Iain 4
Suckling Colin 4
Tucker Nicholas 4 *
1 Heriot-Watt University , Edinburgh , United Kingdom
2 University of Glasgow , Glasgow , United Kingdom
3 University of Birmingham , Birmingham , United Kingdom
4 University of Strathclyde , Glasgow , United Kingdom
*Correspondence: Nicholas Tucker, nick.tucker@strath.ac.uk

Genomics for rational autogenous vaccine design to control Campylobacter infection in poultry

http://jmmcr.microbiologyresearch.org

Calland Jessica *
Mourkas Evangelos
Pascoe Ben
Sheppard Sam
The Milner Centre for Evolution, University of Bath , Bath , United Kingdom
*Correspondence: Jessica Calland, j.k.calland@bath.ac.uk

Combating and detecting Histomonas meleagridis, the causative agent of black head disease, through the administration of novel antimicrobial peptides derived from microbiomes

http://jmmcr.microbiologyresearch.org

Pickup James 1 *
Oyama Linda Dr 1
Nixey Cliff 2
Huws Sharon Dr 1
1 Queens university Belfast , Belfast , United Kingdom
2 British Poulty council , London , United Kingdom
*Correspondence: James Pickup, jablespickup@gmail.com

The bacterial microbiome of in vitro cultures of Paramoeba perurans

http://jmmcr.microbiologyresearch.org

MacPhail David 1 *
Koppenstein Rhea 2
Longshaw Matt 3
Henriquez Fiona 4
1 University of the West of Scotland , Paisley , United Kingdom
2 Hochschule Furtwangen University , Schwarzwald , Germany
3 Benchmark Animal Health , Edinburgh , United Kingdom
4 University of the West of Scotland , Paisley , United Kingdom
*Correspondence: David MacPhail, david.macphail@uws.ac.uk

Novel insights into human cytomegalovirus gene function from a multiplexed proteomic screen of multiple block deletion viruses

http://jmmcr.microbiologyresearch.org

Nightingale Katie 1 *
Fielding Ceri 2
Davies Colin 1
Wang Eddie 2
Antrobus Robin 1
Davison Andrew 3
Wilkinson Gavin 2
Stanton Richard 2
Tomasec Peter 2
Weekes Michael 1
1 University of Cambridge , Cambridge , United Kingdom
2 Cardiff University , Cardiff , United Kingdom
3 University of Glasgow , Glasgow , United Kingdom
*Correspondence: Katie Nightingale, kln25@cam.ac.uk

Rational design of TFDs as novel oligonucleotide antimicrobials to treat Gram-negative infections

http://jmmcr.microbiologyresearch.org

Bahia Sandeep 1
Morris Christopher 1
Laurence Louis 2
McArthur Michael 2 3 *
1 School of Pharmacy, University of East Anglia , Norwich , United Kingdom
2 Norwich Medical School, University of East Anglia , Norwich , United Kingdom
3 Procarta Biosystems , Norwich , United Kingdom
*Correspondence: Michael McArthur, mmcarthur@procartabio.com

Molecular typing of human enteroviruses: reanalysis of previously untypable EV isolates and the rapid detection of EV-D68

http://jmmcr.microbiologyresearch.org

Howson-Wells Hannah 1 *
McClure Patrick 2
Clark Gemma 1
Irving William 2 1
Kidd I. Michael 1 3
1 Nottingham University Hospitals NHS Trust , Nottingham , United Kingdom
2 University of Nottingham , Nottingham , United Kingdom
3 University Hospital Birmingham NHS Foundation Trust , Birmingham , United Kingdom
*Correspondence: Hannah Howson-Wells, hannah.howsonwells@nuh.nhs.uk

Wombling surplus diagnostic nucleic acid for novel pathogenesis and genetic epidemiology of viral infections

http://jmmcr.microbiologyresearch.org

McClure Patrick 1 *
Clark Gemma 2
Chellapuri Akhil 1
Smitheman Matthew 1
Bagasi Arwa 1
Akagha Terry 1
Khandaker Tasneem 1
Howson-Wells Hannah 2
Chappell Joseph 1
Tsoleridis Theocharis 1
Ball Jonathan 1
Irving William 1 2
1 University of Nottingham , Nottingham , United Kingdom
2 Nottingham University Hospitals NHS Trust , Nottingham , United Kingdom
*Correspondence: Patrick McClure, patrick.mcclure@nottingham.ac.uk

High susceptibility, viral dynamics and persistence of South American Zika virus in New World monkeys

http://jmmcr.microbiologyresearch.org

Berry Neil *
Ham Claire
Hall Jo
Jenkins Adrian
Giles Elaine
Kempster Sarah
Rose Nicola
Ferguson Debbie
Almond Neil
NIBSC , South Mimms , United Kingdom
*Correspondence: Neil Berry, neil.berry@nibsc.org

Determining the function of proteins degraded by Human Cytomegalovirus

http://jmmcr.microbiologyresearch.org

Fletcher-Etherington Alice *
Nightingale Katie
Lin Kai-Min
Ravenhill Benjamin
Nobre Luis
Weekes Michael
Cambridge Institute for Medical Research , Cambridge , United Kingdom
*Correspondence: Alice Fletcher-Etherington, af565@cam.ac.uk

Industrially useful microbial species for production of biofuels and chemicals from municipal solid waste

http://jmmcr.microbiologyresearch.org

Dornau Aritha *
Thomas Gavin
McQueen-Mason Simon
University of York , York , United Kingdom
*Correspondence: Aritha Dornau, ad749@york.ac.uk

Enterobacteriaceae-selective modification of the intestinal microbiota using oligonucleotide antimicrobials

http://jmmcr.microbiologyresearch.org

Wong Nichola 1
McArthur Michael 1 2 *
1 Norwich Medical School, University of Eas Anglia , Norwich , United Kingdom
2 Procarta Biosystems , Norwich , United Kingdom
*Correspondence: Michael McArthur, mmcarthur@procartabio.com

Evaluating the epizootic risk to poultry of a novel Chinese H7N9 virus variant with increased pathogenicity in turkeys

http://jmmcr.microbiologyresearch.org

James Joe *
Thomas Saumya
Mahmood Sahar
Seekings Amanda
Slomka Marek
Brown Ian
Brookes Sharon
Animal and Plant Health Agency , Weybridge , United Kingdom
*Correspondence: Joe James, joe.james@apha.gov.uk

The chitin attenuator: the Ca2+/calcineurin pathway maintains the viability of Candida albicans cells with supra-normal chitin levels

http://jmmcr.microbiologyresearch.org

da Silva Dantas Alessandra 1
Gow Neil 1 *
Walker Louise 2
1 University of Exeter , Exeter , United Kingdom
2 University of Aberdeen , Aberdeen , United Kingdom
*Correspondence: Neil Gow, n.gow@exeter.ac.uk

The secretome profiling of a pediatric airway epithelium infected with human respiratory syncytial virus (hRSV) identified aberrant apical/basolateral trafficking and novel immune modulating (CXCL6, CXCL16, CSF3) and antiviral (CEACAM1) proteins

http://jmmcr.microbiologyresearch.org

Touzelet Olivier 1 2 *
Broadbent Lindsay 1
Armstrong Stuart 2 3
Aljabr Waleed 2 4
Cloutman-Green Elaine 5
Power Ultan 1
Hiscox Julian 2 3 6
1 Queen’s University Belfast; School of Medicine, Dentistry & Biomedical Sciences; Wellcome-Wolfson Institute for Experimental Medicine , Belfast , United Kingdom
2 University of Liverpool; Institute of Infection and Global Health; Department of Infection Biology , Liverpool , United Kingdom
3 University of Liverpool; NIHR Health Protection Research Unit in Emerging and Zoonotic Infections , Liverpool , United Kingdom
4 King Fahad Medical City; Research Centre; Biomedical Research Administration , Riyadh , Saudi Arabia
5 Great Ormond Street Hospital; Level 4 Camelia Botnar Laboratory; Microbiology, Virology and Infection Control , London , United Kingdom
6 Agency for Science, Technology and Research (A*STAR); Singapore Immunology Network , Singapore , Singapore
*Correspondence: Olivier Touzelet, o.touzelet@qub.ac.uk

Clinical evaluation of herPHEgen®: a herpesvirus genotype-to-phenotype antiviral resistance database and testing service

http://jmmcr.microbiologyresearch.org

Mohamed Hodan 1
Bibby David 1
Gallagher Eileen 1
Piorkowska Renata 1
Gavriel Andros 1
Manso Carmen 1
Wallis Nigel 1
Parry Chris 1
Zuckerman Mark 2
Cane Patricia 1
Bradshaw Daniel 1 *
Mbisa Jean 1
1 Public Health England , London , United Kingdom
2 King’s College Hospital NHS Foundation Trust , London , United Kingdom
*Correspondence: Daniel Bradshaw, daniel.bradshaw@phe.gov.uk

Direct proteolytic control of an extracytoplasmic function RNA polymerase sigma factor

http://jmmcr.microbiologyresearch.org

Bilyk Bohdan 1 *
Kim Sora 2
Baker Tania 2
Seipke Ryan 1
1 Faculty of Biological Sciences, Astbury Centre for Structural Molecular Biology, University of Leeds , Leeds , United Kingdom
2 Department of Biology, Massachusetts Institute of Technology, Howard Hughes Medical Institute , Cambridge , USA
*Correspondence: Bohdan Bilyk, blbilyk@gmail.com

Use of Nano- SIMS at the single cell-level to evaluate drug penetration into mycobacterial biofilms

http://jmmcr.microbiologyresearch.org

Akwani Winifred 1 2 *
Hingley-Wilson Suzie 1
Chambers Mark 1
Rakowska Paulina 2
McMahon Greg 2
1 University of Surrey , Guildford , United Kingdom
2 National Physical Laboratory , Teddington , United Kingdom
*Correspondence: Winifred Akwani, w.akwani@surrey.ac.uk

The Candida albicans arginase family encodes enzymes with diverse catabolic activities that differentially influence host-fungus interactions

http://jmmcr.microbiologyresearch.org

Schaefer Katja 1
Bates Steven 1
Ames Ryan 1
Christou Stella 2
Gow Neil 1 *
1 Unversity of Exeter , Exeter , United Kingdom
2 University of Aberdeen , Aberdeen , United Kingdom
*Correspondence: Neil Gow, n.gow@exeter.ac.uk

Single cell and single molecule resolution of herpesvirus genome transport, condensation state and transcriptional output

http://jmmcr.microbiologyresearch.org

Sekine Eiki
O’ Hare Peter *
Imperial College , London , United Kingdom
*Correspondence: Peter O’Hare, pohare@imperial.ac.uk

Host response of Agaricus bisporus to mushroom virus X infection

http://jmmcr.microbiologyresearch.org

O’Connor Eoin 1 *
Fitzpatrick David 1
Grogan Helen 2
1 Maynooth University , Maynooth , Ireland
2 Teagasc Food Research Centre , Dublin 15 , Ireland
*Correspondence: Eoin O’Connor, eoin.oconnor@mu.ie

The effect of dietary fatty acid supplementation on gut microbiome development in weaning piglets

http://jmmcr.microbiologyresearch.org

McKenna Robyn 1 2 *
McAdam Paul 2
Smyth Victoria 2
Robinson Mark 1
Cosby Louise 2 1
1 Queen’s University Belfast , Belfast , United Kingdom
2 Agri-Food and Bioscience Institute , Belfast , United Kingdom
*Correspondence: Robyn McKenna, rmckenna24@qub.ac.uk

An investigation of the molecular and cellular mechanisms of in vitro phage therapy in human cells

http://jmmcr.microbiologyresearch.org

Møller-Olsen Christian *
Ho Siu
Ross Toby
Sagona Antonia
School of Life Sciences, University of Warwick , Coventry , United Kingdom
*Correspondence: Christian Moeller-Olsen, c.moller-olsen@warwick.ac.uk

Development of Bacteroides thetaiotaomicron outer membrane vesicles as a universal, mucosal vaccine for Influenza A virus

http://jmmcr.microbiologyresearch.org

Bentley Eleanor 1 *
Al Katy Sanaria 1 2
Kipar Anja 3
Stenz Regis 4
Carding Simon R 4 5
Stewart James 1
1 Institute of Infection and Global Health, University of Liverpool , Liverpool , United Kingdom
2 Department of Pathology and Poultry Diseases, College of Veterinary Medicine, University of Mosul , Mosul , Iraq
3 Institute of Veterinary Pathology, University of Zurich , Zurich , Switzerland
4 Gut Microbes and Health Research Programme, Quadram Institute Bioscience , Norwich , United Kingdom
5 Norwich Medical School, University of East Anglia , Norwich , United Kingdom
*Correspondence: Eleanor Bentley, hlebentl@liverpool.ac.uk

Investigating the role of low-oxygen-activated (lxa) encoded proteins in the pathogenesis of Burkholderia cepacia complex and their contribution to chronic infection in cystic fibrosis

http://jmmcr.microbiologyresearch.org

O’Connor Andrew *
McClean Siobhán
University College Dublin , Dublin , Ireland
*Correspondence: Andrew O’Connor, andrew.oconnor@ucdconnect.ie

Deciphering adaptation through mobile element pangenome composition

http://jmmcr.microbiologyresearch.org

Waters Nicholas 1 2 *
Holmees Ashleigh 2
Abram Florence 1
Pritchard Leighton 2
Brennan Fiona 1 3
1 National University of Ireland , Galway , Ireland
2 The James Hutton Institute , Dundee , United Kingdom
3 Teagasc , Johnstown Castle , Ireland
*Correspondence: Nicholas Waters, n.waters4@nuigalway.ie

Low prevalence of resistance mutations to integrase inhibitors (INI) in patients living with HIV-1 at baseline using next generation sequencing (NGS)

http://jmmcr.microbiologyresearch.org

Hubb Jon *
Hopkins Mark
Deayton Jane
May Sophie
Barts Health NHS Trust , London , United Kingdom
*Correspondence: Jonathan Hubb, jon.hubb@gmail.com

Mycovirus induced hypervirulence of Leptosphaeria biglobosa enhances systemic acquired resistance to Leptosphaeria maculans in Brassica napus

http://jmmcr.microbiologyresearch.org

Shah Unnati 1
Kotta-Loizou Ioly 2 *
Fitt Bruce 1
Coutts Robert 3
1 University of Hertfordshire , Hatfield , United Kingdom
2 Imperial College London , London , United Kingdom
3 University of Hertfordshire , London , United Kingdom
*Correspondence: Ioly Kotta-Loizou, i.kotta-loizou13@imperial.ac.uk

A CRISPR-associated Rossmann fold (CARF) domain regulates transcription of an RNA repair system in Escherichia coli

http://jmmcr.microbiologyresearch.org

Kotta-Loizou Ioly 1 *
Jovanovic Milija 1
Schaefer Jorrit 1
Engl Christoph 2
Zhang Nan 3
Buck Martin 1
1 Imperial College London , London , United Kingdom
2 Queen Mary University of London , London , United Kingdom
3 Houston Methodist Research Institute , Houston , USA
*Correspondence: Ioly Kotta-Loizou, i.kotta-loizou13@imperial.ac.uk

Use of Bacteroides-derived microvesicles for mucosal vaccines

http://jmmcr.microbiologyresearch.org

Miquel-Clopes Ariadna 1 *
Carvalho Ana L. 1
Wegmann Udo 1
Bentley Eleanor G. 2
Al-Katy Sanaria H.M. 2
Coombes Janine L. 2
Kipar Anja 3
Stentz Regis 1
Stewart James P. 2
Carding Simon R. 1 4
1 Quadram Institute Bioscience , Norwich , United Kingdom
2 University of Liverpool , Liverpool , United Kingdom
3 Instiute of Veterinary Pathology , Zurich , Switzerland
4 University of East Anglia , Norwich , United Kingdom
*Correspondence: Ariadna Miquel-Clopes, ariadna.miquel-clopes@quadram.ac.uk

Identification of niche-specific virulence factors via experimental evolution of Streptococcus pneumoniae

http://jmmcr.microbiologyresearch.org

Green Angharad *
Neill Daniel
Hinton Jay
University of Liverpool , Liverpool , United Kingdom
*Correspondence: Angharad Green, angharad.green@liverpool.ac.uk

Using cryo-electron tomography to elucidate the structural basis of the endosomal K+ requirement during Bunyavirus entry

http://jmmcr.microbiologyresearch.org

Hover Samantha *
Punch Emma
Fontana Juan
Barr John
Mankouri Jamel
University of Leeds , Leeds , United Kingdom
*Correspondence: Samantha Hover, bs11s3h@leeds.ac.uk

Developing a universal strategy for cloning and assembly of the genomes of diverse Epstein – Barr virus strains

http://jmmcr.microbiologyresearch.org

Bayoumy Amr *
Abdullah Mohammed Ba
White Rob
Imperial College London , London , United Kingdom
*Correspondence: Amr Bayoumy, amrvet99@yahoo.com

Characterising the genomes and transcriptomes of hyper ammonia producing bacteria from the rumen

http://jmmcr.microbiologyresearch.org

Friedersdorff Jess 1 *
Creevey Chris 2
Kingston-Smith Alison 1
1 Aberystwyth University , Aberystwyth , United Kingdom
2 Queen’s University , Belfast , United Kingdom
*Correspondence: Jess Friedersdorff, jef11@aber.ac.uk

Investigating evolution of the paediatric cystic fibrosis lung microbiota using induced sputum sampling and culture-independent techniques

http://jmmcr.microbiologyresearch.org

Weiser Rebecca 1 *
Oakley Juliette 2
Jones Cerith 3
Mahenthiralingam Eshwar 1
Forton Julian 2 4
1 Cardiff University, Cardiff School of Biosciences , Cardiff , United Kingdom
2 Children’s Hospital for Wales, Department of Paediatric Respiratory Medicine , Cardiff , United Kingdom
3 University of South Wales, School of Applied Sciences, Faculty of Computing, Engineering and Science , Pontypridd , United Kingdom
4 Cardiff University, School of Medicine , Cardiff , United Kingdom
*Correspondence: Rebecca Weiser, weiserr@cf.ac.uk

African Horse Sickness virus: pathogenicity in an IFNAR(-/-) mouse model of infection

http://jmmcr.microbiologyresearch.org

Jones Luke 1 *
Hawes Pippa 2
Salguero Javier 3
Castillo-Olivares Javier 4 5
1 The Pirbright Institute , Pirbright , United Kingdom
2 Pirbright Institute , Pirbright , United Kingdom
3 Public Health England , Porton Down , United Kingdom
4 University of Cambridge , Cambridge , United Kingdom
5 University of Surrey , Guildford , United Kingdom
*Correspondence: Luke Jones, lj00260@surrey.ac.uk

Applying Next Generation Sequencing Technology to Hepatitis C genotyping in a routine diagnostic laboratory

http://jmmcr.microbiologyresearch.org

May Sophie *
Barts Health Trust , London , United Kingdom
*Correspondence: Sophie May, sophie.may@bartshealth.nhs.uk

Identification of novel factors associated with severe respiratory syncytial virus disease in infants

http://jmmcr.microbiologyresearch.org

Parke Hong Guo 1
Groves Helen 1
Broadbent Lindsay 1
Touzelet Olivier 1
Douglas Isobel 2
Campos Guillermo Lopez 1
Canning Paul 1
Lyons Jeremy D. 2
Coyle Peter 3
Heaney Liam 1
Shields Michael D. 1
Power Ultan F. 1 *
1 Wellcome Wolfson Institute for Experimental Medicine, Queen’s University Belfast , Belfast , United Kingdom
2 The Royal Belfast Hospital for Sick Children , Belfast , United Kingdom
3 The Regional Virus Laboratory, Belfast Trust , Belfast , United Kingdom
*Correspondence: Ultan Power, u.power@qub.ac.uk

Characterising the insect-borne transmission of the cattle poxvirus lumpy skin disease virus

http://jmmcr.microbiologyresearch.org

Sanz-Bernardo Beatriz *
Wijesiriwardana Najith
Haga Ismar
Gubbins Simon
Hawes Pippa
Wilson Anthony
Sanders Christopher
Basu Sanjay
Alphey Luke
Langlands Zoe
Darpel Karin
Beard Pip
The Pirbright Institute , Woking , United Kingdom
*Correspondence: Beatriz Sanz Bernardo, beatriz.sanz-bernardo@pirbright.ac.uk

The long isoform of ZAP widely restricts Paramyxoviruses

http://jmmcr.microbiologyresearch.org

Thibault Patricia A. 1 *
Ryan Andrew P. 2
Hung Chuan-tien 1
Daugherty Matthew 2
Lee Benhur 1
1 Icahn School of Medicine at Mount Sinai , New York , USA
2 University of California , San Diego, La Jolla , USA
*Correspondence: Patricia Thibault, PATRICIA.THIBAULT@MSSM.EDU

Tracking plasmid-mediated antibiotic resistance from environmental reservoirs to the food chain

http://jmmcr.microbiologyresearch.org

Murphy Sinéad 1 *
Joyce Aoife 1
Vollenweider Vera 2
Drissner David 2
Walsh Fiona 1
1 Maynooth University , Maynooth, Co. Kildare , Ireland
2 Agroscope , Zurich , Switzerland
*Correspondence: Sinéad Murphy, murphy.sineadm7@gmail.com

Spatiotemporal dynamics of host cell modification caused by herpesvirus infection

http://jmmcr.microbiologyresearch.org

Scherer Katharina M. 1 *
Soh Timothy 2
Manton James D. 3
Mascheroni Luca 1
Crump Colin M. 1
Kaminski Clemens F. 1
1 University of Cambridge , Cambridge , United Kingdom
2 Heinrich-Pette-Institut , Hamburg , Germany
3 Laboratory of Molecular Biology , Cambridge , United Kingdom
*Correspondence: Katharina Scherer, ks820@cam.ac.uk

Cellular cholesterol abundance regulates potassium accumulation within endosomes and is an important determinant in Bunyavirus entry

http://jmmcr.microbiologyresearch.org

Charlton Frank 1 *
Hover Samantha 1
Fuller Jack 1
Hewson Roger 2
Fontana Juan 3
Barr John 3
Mankouri Jamel 3
1 School of Molecular and Cellular Biology, University of Leeds , Leeds , United Kingdom
2 National Infection Service, Public Health England , Porton Down, Salisbury , United Kingdom
3 Astbury Centre for Structural Molecular Biology, University of Leeds , Leeds , United Kingdom
*Correspondence: Frank Charlton, frankwcharlton@gmail.com

Investigation and genome characterisation of tatenale hantavirus in wild rodent populations in the United Kingdom

http://jmmcr.microbiologyresearch.org

Chappell Joseph G *
Tsoleridis Theocharis
McClure C. Patrick
Bennett Malcolm
Tarlinton Rachael E
Ball Jonathan K
University of Nottingham , Nottingham , United Kingdom
*Correspondence: Joe Chappell, nixjc1@nottingham.ac.uk

High-throughput sequencing of patients with symptoms of unknown Etiology

http://jmmcr.microbiologyresearch.org

Chappell Joseph G 1 *
Tsoleridis Theocharis 1
McClure C. Patrick 1
Tarlinton Rachael 1
Clark Gemma 2
Irving William L 1 2
Ball Jonathan K 1
1 University of Nottingham , Nottingham , United Kingdom
2 Nottingham University Hospitals NHS Trust , Nottingham , United Kingdom
*Correspondence: Joe Chappell, nixjc1@nottingham.ac.uk

The Savage Dawn Peptide: an antibiotic woven from from 12th century Welsh poetry

http://jmmcr.microbiologyresearch.org

Thomas Benjamin 1 2 *
Gani Jurnorain 3
Creevey Chris 2
Hilpert Kai 3
1 Aberystwyth University , Aberystwyth , United Kingdom
2 Queen’s University , Belfast , United Kingdom
3 St. George’s Hospital , London , United Kingdom
*Correspondence: Ben Thomas, bet16@aber.ac.uk

Induction of inflammasome-dependent signalling in the human monocytic cell line THP-1 by Campylobacter lipooligosaccharides

http://jmmcr.microbiologyresearch.org

Hameed Amber 1
Machado Lee 1
Woodacre Alexandra 1 *
Marsden Gemma 2 1
1 University of Northampton , Northampton , United Kingdom
2 Hospital Infection Society , London , United Kingdom
*Correspondence: Alexandra Woodacre, alexandra.woodacre@northampton.ac.uk

Investigating the interferon antagonistic abilities of louping ill virus, a neglected animal pathogen endemic to the UK

http://jmmcr.microbiologyresearch.org

Clark Jordan 1 2 *
Goertz Giel 3
Schnettler Esther 4
McInnes Colin 2
Biek Roman 5
Kohl Alain 1
1 MRC – University of Glasgow Centre for Virus Research , Glasgow , United Kingdom
2 Moredun Research Institute , Penicuik , United Kingdom
3 Wageningen University & Research , Wageningen , Netherlands
4 Bernhard Nocht Institute for Tropical Medicine , Hamburg , Germany
5 Institute of Biodiversity, Animal Health & Comparative Medicine, University of Glasgow , Glasgow , United Kingdom
*Correspondence: Jordan Clark, jordan.clark@liverpool.ac.uk

Deciphering the secretion mechanism and novel protein-protein interactions of TecA, a Burkholderia cenocepacia toxin

http://jmmcr.microbiologyresearch.org

Monjaras-Feria Julia *
Bisaro Fabiana
Valvano Miguel
Queen’s University of Belfast , Belfast , United Kingdom
*Correspondence: Julia Monjaras-Feria, J.MonjarasFeria@qub.ac.uk

Swine influenza A viruses with zoonotic potential – PCR HA/NA typing, and differential detection of pandemic09 reassortants in GB and European pigs

http://jmmcr.microbiologyresearch.org

Brookes Sharon 1 *
Reid Scott 1
Lewis Nicola 1
Chepkwony Sharon 2
Russell Christine 1
Cooper Jayne 1
Coward Vivien 1
Everett Helen 1
Coney Elizabeth 1
Byrne Alexander 1
Parys Anna 2
Vandoorn Elien 2
Byrne Dominic 1
Williamson Susanna 3
Van Reeth Kristien 2
Brown Ian 1
1 APHA-Weybridge , New Haw , United Kingdom
2 Ghent University , Ghent , Belgium
3 APHA-Bury , Bury St Edmunds , United Kingdom
*Correspondence: Sharon Brookes, sharon.brookes@apha.gov.uk

The effects of APOBEC3 proteins on Hepatitis B virus replication

http://jmmcr.microbiologyresearch.org

Elbusifi Ibrahim *
McGarvey Michael
Imperial College London , London , United Kingdom
*Correspondence: Ibrahim Elbusifi, i.elbusifi14@imperial.ac.uk

Viral adaptation to alternating hosts and associated allelic variants

http://jmmcr.microbiologyresearch.org

Ayansola Oyeronke *
Dickins Benjamin
McVicker Gareth
Loughlin Michael
Nottingham Trent University , Nottingham , United Kingdom
*Correspondence: Oyeronke Ayansola, ronkay2008@hotmail.co.uk

Replicative fitness and transmission of G57 lineage and UDL01 like H9N2 viruses in chickens

http://jmmcr.microbiologyresearch.org

Bhat Sushant *
Shelton Holly
Sadeyen Jean-Remy
Iqbal Munir
The Pirbright Institute , Woking , United Kingdom
*Correspondence: Sushant Bhat, sushant.bhat@pirbright.ac.uk

Aedes Aegypti(Aag2)-derived clonal mosquito cell lines reveal impact of pre-existing persistent co-infection with the insect-specific Bunyavirus Phasi Charoen-Like Virus on Arbovirus replication

http://jmmcr.microbiologyresearch.org

Wallace Louisa 1
Fredericks Anthony 2
Bernal-Rubio Dabeiba 2
Davidson Andrew 3
Fernandez-Sesma Ana 2
Maringer Kevin 1 *
1 University of Surrey , Guildford , United Kingdom
2 Icahn School of Medicine at Mount Sinai , New York , USA
3 University of Bristol , Bristol , United Kingdom
*Correspondence: Kevin Maringer, k.maringer@surrey.ac.uk

Retrospective and prospective evaluation of chickenpox post-exposure prophylaxis (PEP) in at-risk groups before and after the change in PEP guidelines

http://jmmcr.microbiologyresearch.org

Harris Stephanie *
Zuckerman Mark
King’s College Hospital , London , United Kingdom
*Correspondence: Stephanie Harris, Stephanie.harris5@nhs.net

In vitro reconstitution of the polymicrobial community associated with cystic fibrosis airway infections

http://jmmcr.microbiologyresearch.org

O’Brien Tom *
Welch Martin
University of Cambridge , Cambridge , United Kingdom
*Correspondence: Tom O’Brien, tjo37@cam.ac.uk

Equally contributing 1st co-authors.

